# Transcriptional signatures associated with persisting CD19 CAR-T cells in children with leukemia

**DOI:** 10.1038/s41591-023-02415-3

**Published:** 2023-07-06

**Authors:** Nathaniel D. Anderson, Jack Birch, Theo Accogli, Ignacio Criado, Eleonora Khabirova, Conor Parks, Yvette Wood, Matthew D. Young, Tarryn Porter, Rachel Richardson, Sarah J. Albon, Bilyana Popova, Andre Lopes, Robert Wynn, Rachael Hough, Satyen H. Gohil, Martin Pule, Persis J. Amrolia, Sam Behjati, Sara Ghorashian

**Affiliations:** 1 Wellcome Sanger Institute, Hinxton, UK; 2 Developmental Biology and Cancer, UCL Great Ormond Street Institute of Child Health, London, UK; 3 Molecular and Cellular Immunology, UCL Great Ormond Street Institute of Child Health, London, UK; 4 Cancer Research UK & UCL Cancer Trials Centre, London, UK; 5 Department of Bone Marrow Transplantation, Royal Manchester Children’s Hospital, Manchester, UK; 6 Children and Young People’s Cancer Service, University College London Hospitals NHS Foundation Trust, London, UK; 7 Department of Haematology, University College London Hospitals NHS Foundation Trust, London, UK; 8 Department of Haematology, UCL Cancer Institute, London, UK; 9 Department of Bone Marrow Transplantation, Great Ormond Street Hospital for Children, London, UK; 10 Department of Paediatrics, University of Cambridge, Cambridge, UK; 11 Cambridge University Hospitals NHS Foundation Trust, Cambridge, UK; 12 Department of Haematology, Great Ormond Street Hospital for Children, London, UK

**Keywords:** Immunotherapy, Paediatric cancer, Acute lymphocytic leukaemia

## Abstract

In the context of relapsed and refractory childhood pre-B cell acute lymphoblastic leukemia (R/R B-ALL), CD19-targeting chimeric antigen receptor (CAR)-T cells often induce durable remissions, which requires the persistence of CAR-T cells. In this study, we systematically analyzed CD19 CAR-T cells of 10 children with R/R B-ALL enrolled in the CARPALL trial via high-throughput single-cell gene expression and T cell receptor sequencing of infusion products and serial blood and bone marrow samples up to 5 years after infusion. We show that long-lived CAR-T cells developed a CD4/CD8 double-negative phenotype with an exhausted-like memory state and distinct transcriptional signature. This persistence signature was dominant among circulating CAR-T cells in all children with a long-lived treatment response for which sequencing data were sufficient (4/4, 100%). The signature was also present across T cell subsets and clonotypes, indicating that persisting CAR-T cells converge transcriptionally. This persistence signature was also detected in two adult patients with chronic lymphocytic leukemia with decade-long remissions who received a different CD19 CAR-T cell product. Examination of single T cell transcriptomes from a wide range of healthy and diseased tissues across children and adults indicated that the persistence signature may be specific to long-lived CAR-T cells. These findings raise the possibility that a universal transcriptional signature of clinically effective, persistent CD19 CAR-T cells exists.

## Abstract (web summary)

In children with relapsed or refractory B cell acute lymphoblastic leukemia and in complete remission after CD19 CAR-T cell therapy, long-lived CAR-T cells express a persistence gene signature that is also present in persistent CD19 CAR-T cells from adults with chronic lymphocytic leukemia.

## Main

B-lineage acute lymphoblastic leukemia (B-ALL) is the most common type of childhood cancer and mostly derives from immature B cells that carry the cell surface antigen CD19 (ref. ^1^). Most children with B-ALL can be cured through first-line treatment comprising combinations of cytotoxic agents. However, relapsed ALL remains a leading cause of childhood death despite intensive cytotoxic chemotherapy often including allogeneic bone marrow transplantation. The advent of CD19 chimeric antigen receptor (CAR)-T cell therapy in recent years has transformed the treatment of intractable ALL^2^. Although a subset of children can be cured, up to 60% of children experience further, typically fatal, disease recurrence due to non-persistence of CAR-T cells or CD19^−^ leukemic escape^3,4^.

Previously, we generated a novel low-affinity CAR incorporating a CD19-specific single-chain variable fragment (scFv) called CAT, displaying a faster off-rate of interaction than the FMC63 CD19 binder used in prior clinical studies^3^. CAT CAR-T cells showed greater cytotoxicity and proliferative responses in vitro and maintained long-lived molecular remissions in children with relapsed or refractory ALL, as demonstrated in the CARPALL study^3^. The molecular features underpinning CAR-T cell persistence in our study remain unknown. We reasoned that single-cell transcriptomic assays may help elucidate these features. To date, other CAR-T cell products in patients have been studied at the resolution of single cells^5,6^. However, the persistence of CAR T-cells in these studies was generally limited to 3 months. An exception was long-lived CAR-T cells in two adult individuals with a different cancer—chronic lymphocytic leukemia (CLL)—in whom anti-CD19 CAR-T cells have persisted for almost a decade thus far^7^. It is unclear whether one can generalize from two adult patients treated for CLL to other hematological malignancies and patient groups, in particular to childhood ALL, or to other CAR-T cell products.

We systematically studied molecular features and clonal dynamics of CAR-T cells in children enrolled in the CARPALL study at serial timepoints, from production to persistence, up to 5 years after infusion.

## Results

### Overview of study cohort and experiment

**Fig. 1:**
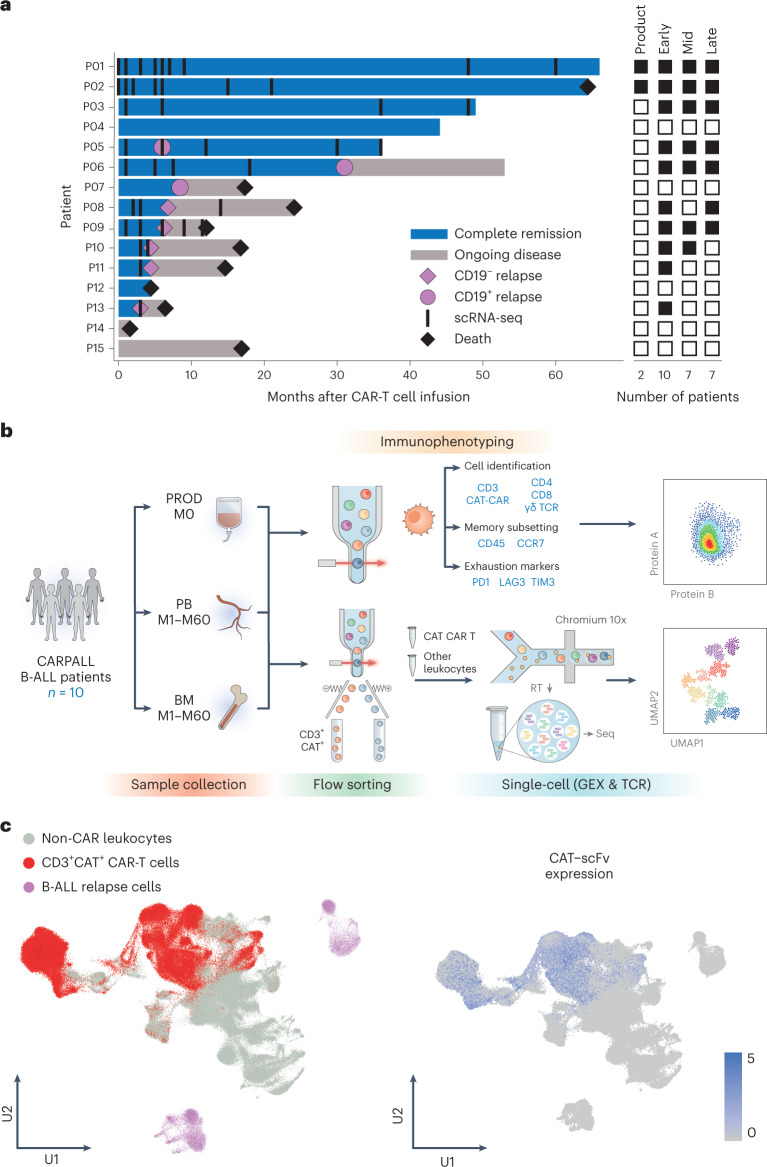
Study overview and workflow. **a**, Swimmer plot illustrating the responses of individual pediatric patients with B-ALL to CAT CAR T-cell therapy and timepoints of sample collection. Attainment of complete remission was associated with attainment of B cell aplasia in all cases. Patient 5 had an isolated unilateral ocular relapse of CD19^+^ leukemia, which was treated with enucleation and remains in ongoing minimal residual disease (MRD) negative remission with no other intervention. Heat map to the right demonstrates timepoint representation per patient. Filled black boxes indicate the presence of the timepoint. Product = infusion products (M0); early = M1–M3; mid = M4–M6; late = M7–M60. **b**, Schematic workflow of study design. Samples were collected from infusion products (PROD), peripheral blood (PB) and bone marrow (BM) between M0 and M60. Samples were used either for flow-based immunophenotyping or for single-cell GEX and TCR sequencing on the Chromium 10x platform. RT, reverse transcription; Seq, sequencing. **c**, UMAP of all cells in the dataset highlighting cell types captured (left) and expression of the CAT-scFv CAR construct (right). CAT-scFv, low-affinity CAR (CAT) incorporating a CD19-specific scFv.

**Extended Data Fig. 1:**
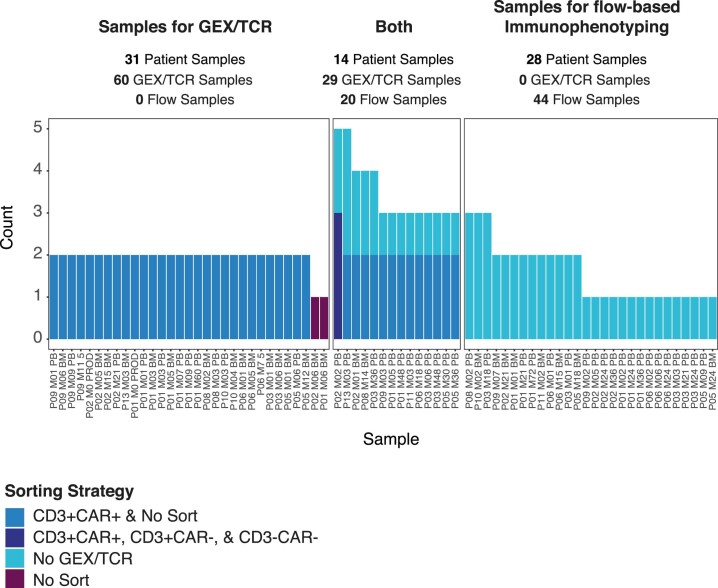
Sample breakdown. Bar charts show the number of experimental samples derived from each patient sample. Each patient sample, defined as an individual, timepoint and sample source (that is P01 M01 PB) can be used for gene expression (GEX) and T-cell receptor (TCR) sequencing and/or flow-based immunophenotyping. For those samples with GEX/TCR sequencing, they are flow-sorted by CD3 and CAR prior to sequencing, with the exception of P02 M02 PB which was sorted for CAR-T cells, T cells and other leukocytes, thus they only contribute 1 experimental sample each. For two samples (P02 M06 BM and P01 M06 BM), no cells were recovered from the CAR T channel. M=month, PB = peripheral blood, BM = bone marrow.

We studied 15 consecutive patients with high-risk or relapsed CD19^+^ B-ALL treated with CD19 CAR-T cell therapy on the CARPALL study (NCT02443831) and in whom adequate CAR-T cells could be isolated for subsequent analyses from cryopreserved samples of blood or bone marrow. Outcomes of the first 14 patients infused were reported^3^; subsequently, a further 18 patients have been treated. Thirteen of 15 (87%) patients studied achieved complete remission; six of these responding patients subsequently relapsed, whereas the other seven achieved long-lived remissions maintained by detectable CAR-T cells and concomitant B cell aplasia (Fig. 1a). We performed detailed phenotyping by flow cytometry in 11 patients, and, in ten children, sufficient CAR-T cells were obtained for further interrogation by single-cell mRNA and T cell receptor (TCR) sequencing (73 patient samples split into 89 gene expression (GEX)/TCR and 64 flow samples; Supplementary Table 1 and Extended Data Fig. 1). Samples were taken from the CAR-T cell product as well as from patients at early (months 1–3), mid (months 4–6) and late (month 7 onwards) timepoints. Early timepoints were defined as 1–3 months, as all patients who achieved molecular complete remission with the absence of measurable residual disease did so within this window. The late timepoints were selected based on the timing of CD19^−^ relapses, which were generally early events and would have occurred by month 7. The mid timepoints were the interval remaining between early and late. Two patients had samples from the product and at all timepoints (early, mid and late); four patients had all post-infusion timepoints represented; two patients had samples at two of three post-infusion timepoints; and two patients had only the early timepoint interrogated due to early relapse. We isolated CAR-T cells from peripheral blood or bone marrow by flow cytometry using CD3 and CAR expression, before single-cell sequencing (Chromium 10x platform) (Fig. 1b).

### Double-negative CAR-T cells delineate late timepoints

**Fig. 2:**
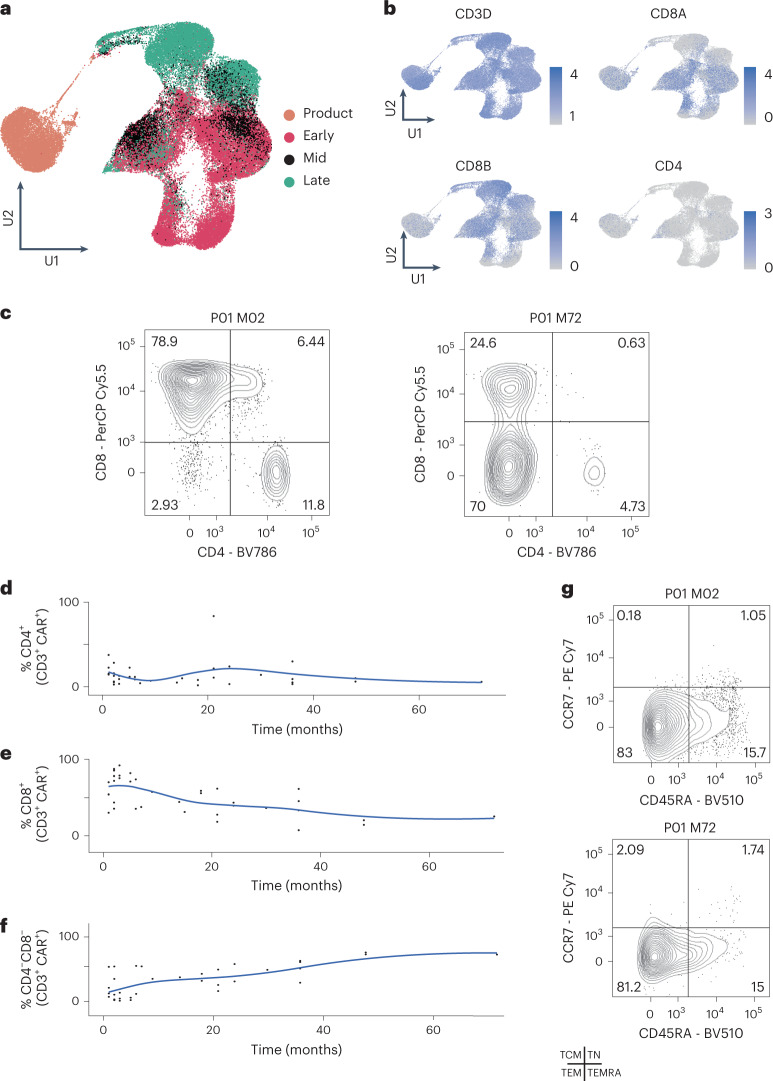
Characterization of CARPALL CAR-T cells. **a**, UMAP of CAT CAR-T cells demonstrates transcriptional clustering based on timepoint. Product = infusion products (M0); early = M1–M3; mid = M4–M6; late = M7–M60. **b**, UMAPs show scaled average expression of lymphocyte markers. **c**, Immunophenotyping CAT CAR-T cells by multi-parameter flow cytometry. Representative example of P01 showing cell identification using CD4 and CD8A at an early (M2) and a late (M72) timepoint. Cells were gated for CD3 and CAT CAR. **d–f**, Trajectory scatter plots quantify data from all patient samples for CD4, CD8 and double-negative CAR-T populations. **g**, Representative example of P01 showing immunophenotyping using CD45RA and CCR7 at an early (M2) and a late (M72) timepoint. Cells were gated for CD3 and CAT CAR. TCM, central memory; TEM, effector memory; TEMRA, terminally differentiated effector memory expressing CD45RA; TN, naive; TSCM, stem cell memory. Fluorochromes: BV, brilliant violet; Cy, cyanine dye; PE, R-phycoerythrin; PerCP, peridinin-chlorophyll protein. Source data

**Extended Data Fig. 2:**
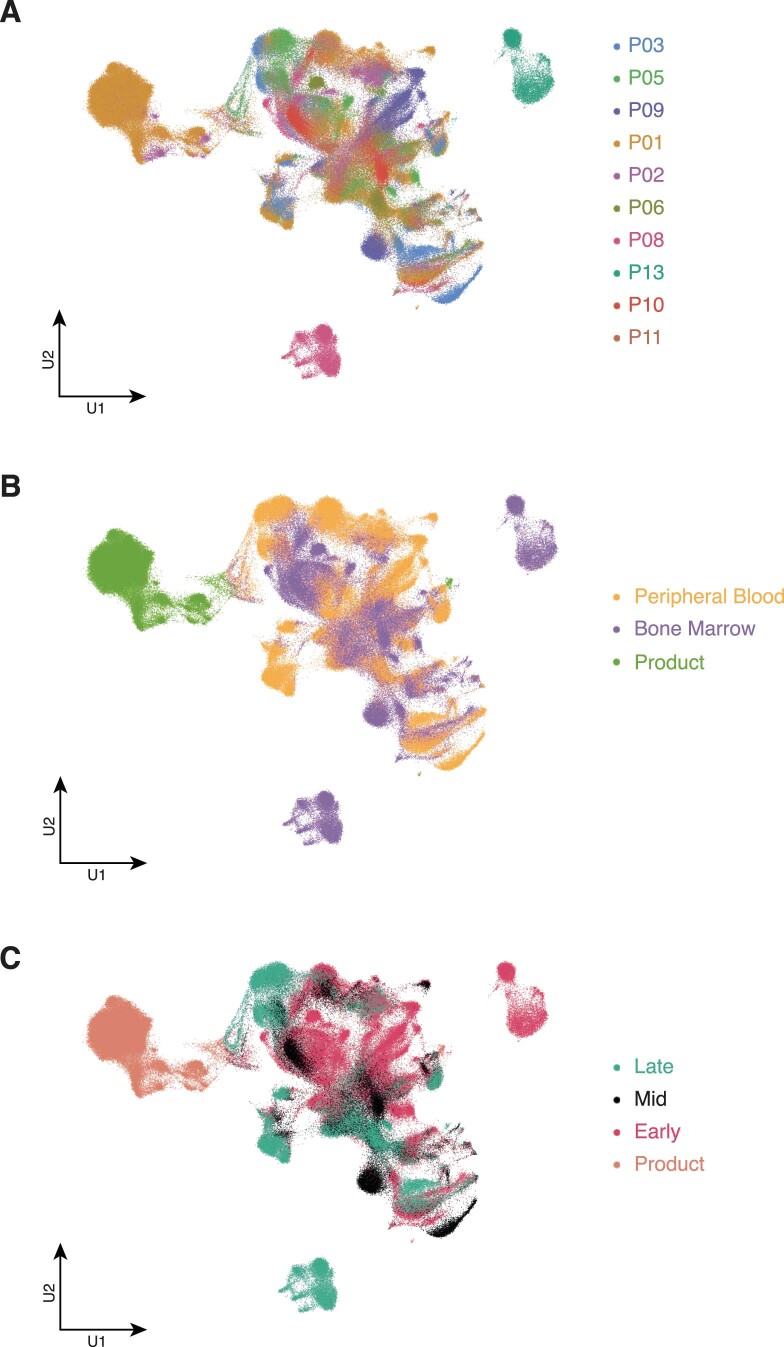
Global cluster annotation. Uniform Manifold Approximation Projections (UMAPs) show all cells in the dataset coloured by **(a)** patient of origin, (**b**) sample source, and **(c)** timepoint.

**Extended Data Fig. 3:**
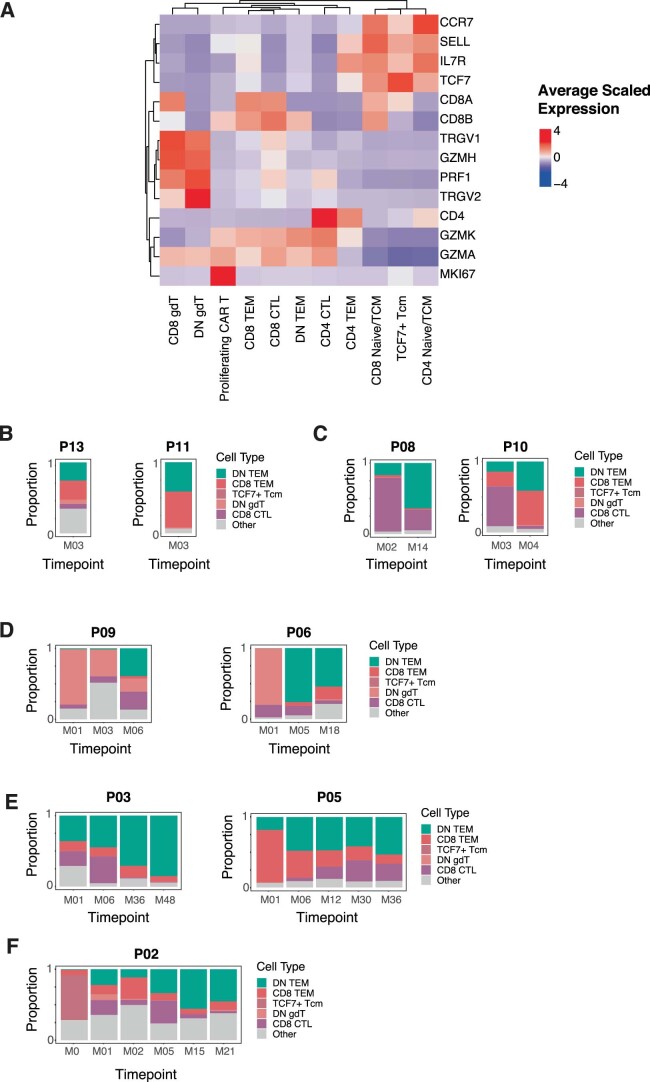
Cluster markers and cell type trajectories. **(a)** Heatmap shows the average scaled expression of marker genes used for cell type annotation. **(b–f)** Stacked bar plots show dynamic cell type proportions over time for all patients. Top 5 abundant cell types in the dataset are shown.

In total, we recovered 264,827 single cells that passed quality control, approximately 50,000 of which were CAR-T cells (Supplementary Table 2). We grouped all 264,827 cells using commonly deployed analytical methods and visualized resultant clusters using uniform manifold approximation and projection (UMAP) (Fig. 1c and Extended Data Fig. 2). Clustering segregated CAR-T cells from non-CAR-T cells, with contributions from all patients. Two clusters were completely patient specific; these clusters represented ALL cancer cells from two children, patients P13 and P08, with CD19^−^ relapses at the time of sampling. Subclustering of CAR-T cells segregated cells transcriptionally into infusion products at month 0 (M0), followed by early (M1–3), mid (M4–M6) and late (M7–M60) timepoints after infusion (Fig. 2a). Cycling cells congregated together from all timepoints, indicating that CAR-T cells remain proliferative several years after infusion. Using a marker-based annotation, CD8^+^ T cells were the predominant CAR-T cell at all timepoints in most cases, apart from late timepoints where CAR-T cells lacked expression of both CD4 and CD8A transcripts (Fig. 2b, Extended Data Fig. 3 and Supplementary Table 3). CD4 CAR-T cells made minor contributions at this point. Thus, late or persisting CAR-T populations were predominantly double-negative T cells.

### Validation of double-negative CAR-T cell state

**Extended Data Fig. 4:**
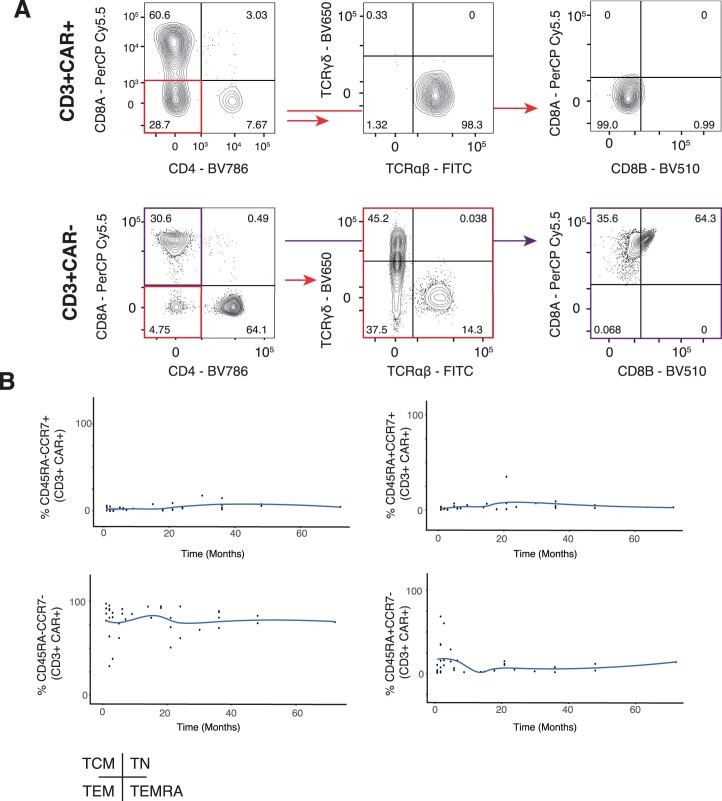
General flow-based immunophenotyping. **(a)** Immunophenotyping non-CAR and CAR-T cells by multi-parameter flow cytometry. All cells were initially gated for CD3 and the CAR. Red boxes and arrows highlight subsequent gating strategies for representative examples illustrating double-negative CAR-T cells are αβ T cells and CD8A/CD8B negative, in contrast to non-CAR-T cells. Fluorochromes: PerCP-Cy5.5 = Peridinin chlorophyll protein-Cyanine5.5, FITC = Fluorescein isothiocyanate, Brilliant Violet (BV) 650, BV510, BV786. **(b)** Trajectory scatter plots quantify flow plots signals across patient samples for CD45RA and CCR7. Cells were gated for CD3 and the CAR. TN = Naïve, TSCM = Stem Cell Memory, TCM = Central Memory, TEM = Effector Memory, TEMRA = Terminally Differentiated Effector Memory expressing CD45RA.

To confirm the early predominance of a CD8^+^ subset and the later emergence of a double-negative population, we implemented two orthogonal approaches. First, we analyzed peripheral blood (PB) and bone marrow (BM) samples from seven CARPALL patients collected at late timepoints by flow cytometry (7–72 months after infusion; Supplementary Table 1). We identified CAR-T cells using CD3 expression and use of an anti-idiotype antibody specific for the CAR and assessed expression of CD4 and CD8 on CAR-T cells (Fig. 2c–f and Extended Data Fig. 4a). This analysis confirmed that most cells were double negative at these late timepoints with a smaller contribution from CD8 T cells. This contrasted with the lower proportion of double-negative T cells in the non-CAR-T cell compartment in these patients (Supplementary Table 4). Furthermore, CAR-T cells were also characterised by lack of expression of CD45RA and CCR7, suggesting an effector memory phenotype (Fig. 2g and Extended Data Fig. 4b).

**Fig. 3:**
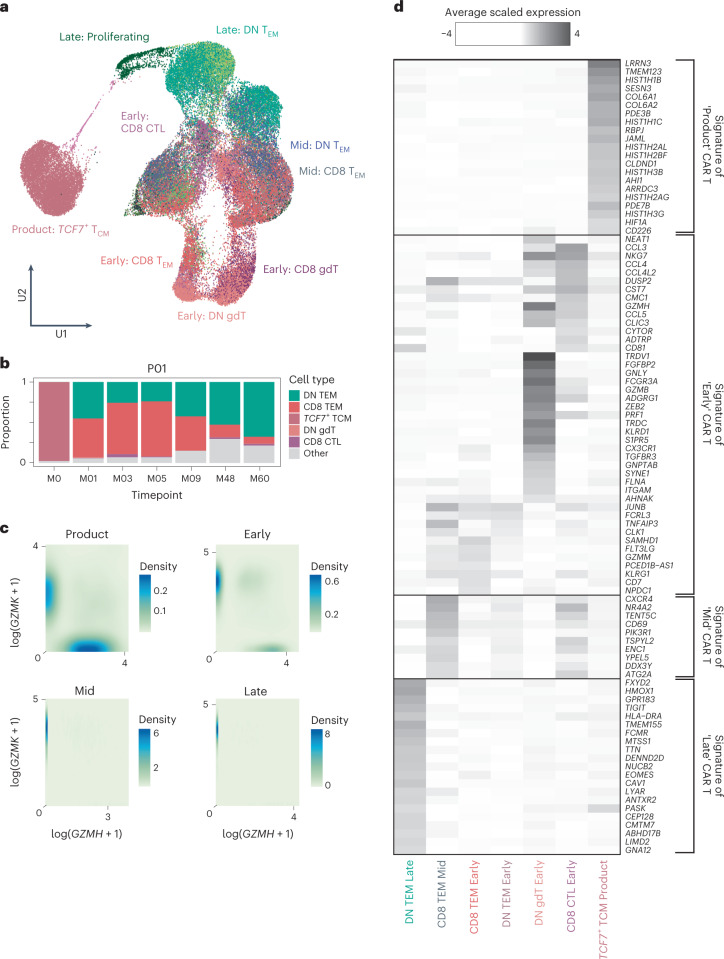
Cell typing and defining a persisting CAR-T cell transcriptional signature. **a**, UMAP of CAT CAR-T cells shows closest cell type matching using a PBMC reference and cell-marker-based annotation. Late (persisting, M7–M60) CAR-T cells are in green hues; mid (M4–6) CAR-T cells are in black/blue hues; and early (M1–3) CAR-T cells are in pink/red hues. **b**, Stacked bar plots show dynamic cell type proportions over time for a representative patient (P01). The top five abundant cell types in the dataset are shown. **c**, Contour plots quantify expression of *GZMK* against *GZMH* across patients per timepoint. **d**, Heat map shows differential gene expression results creating gene signatures of each timepoint. DN, double-negative; TCM, central memory; TEM, effector memory.

**Extended Data Fig. 5:**
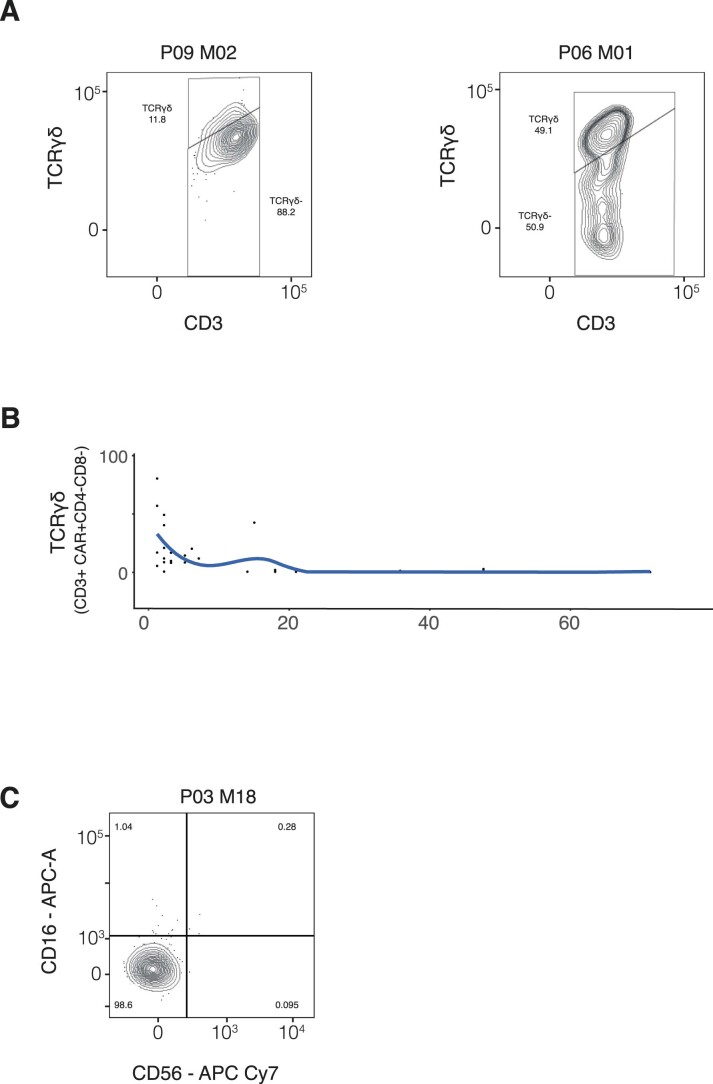
Gamma-Delta and NK Immunophenotyping CAR-T cells by multi-parameter flow cytometry. All cells were initially gated for CD3, CAR, CD4 and CD8A. **(a)** Flow plots for representative samples (P06 and P09) with high populations of double-negative TCRγδ cells. **(b)** Trajectory scatter plots quantify flow plots signals across patient samples for TCRγδ. Cells were previously gated for CD3, CAR, CD4 and CD8. **(c)** Flow plots show gating for CD16 and CD56. Prior gating includes CD3, CAR, CD4, CD8. Fluorochromes: APC = Allophycocyanin, Cy = Cyanin.

In our second approach, we pursued a cell-marker-independent analysis to assign cell identity to CAR-T cells. We directly compared CAR-T cell transcriptomes to a multi-modal, single-cell atlas of the circulating human immune system^8^. This reference is based on 211,000 human blood mononuclear cells interrogated by single-cell mRNA sequencing and by 228 anti-surface protein antibodies (CITE-seq). Consistent with our initial annotation and protein validation by flow cytometry, we observed that most late-persisting CAR-T cells were classified as double-negative cells, whereas CAR-T cells from earlier timepoints were mainly CD8^+^ T cells (Fig. 3a,b). The exceptions were patients P09 and P06 in whom an appreciable quantity of early CAR-T cells were double-negative γδ T cells with high expression of *NKG7* and *GNLY* (Extended Data Fig. 5). This is consistent with a previous report that γδ T lymphocytes harbor similarities to CD8 T cells and natural killer (NK) cells^9^. Together, our initial observation with validation by two approaches demonstrates that most persisting CAR-T cells represented double-negative αβ T cells.

### Persisting CAR-T cells exhibit a transcriptional signature

**Extended Data Fig. 6:**
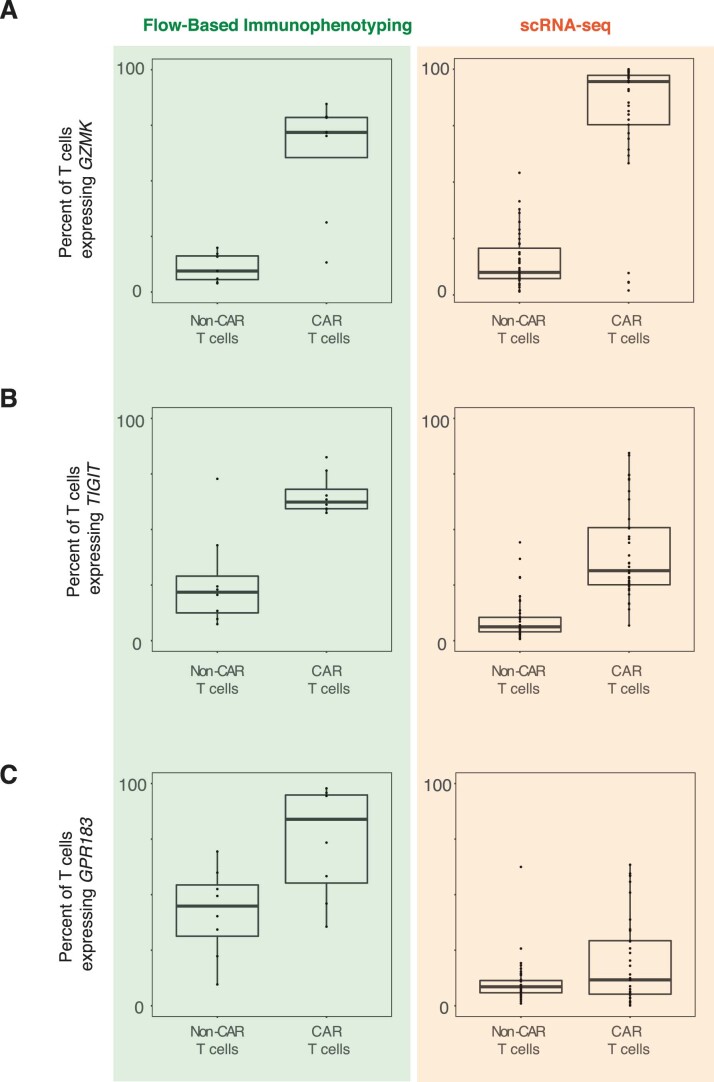
Expression of key genes in the persisting CAR T-cell signature. Boxplots show the percent of T cell expression for **(a)** *GZMK*, **(b)** *TIGIT*, and **(c)** *GPR183* for flow-based immunophenotyping (left) and single-cell RNA sequencing (scRNA-seq) data. CAR-T cell scRNA-seq, data is derived from 60188 cells from 43 independent samples. For non-CAR T-cell scRNAseq, data is derived from 98003 cells from 43 independent samples. Flow data is derived from 15 independent samples. Box plots show the first quartile (the lower end of the box) and the third quartile (the upper other end of the box), as well as the median values (centre line) per dataset. The ‘whiskers’ extend from the ends of the box to a maximum and minimum of 1.5 times the interquartile range beyond the box. Outliers are shown as dots.

Next, we identified differentially expressed genes among CAR-T cells from each timepoint to extract markers of infusion, early, mid and late CAR-T cells. Infusion products were enriched for genes related to cell cycle, nucleosome assembly and glycolysis, plausibly due to in vitro activation during manufacture. Infusion products expressed high levels of genes reflecting naive lymphocyte (that is, *SELL*, *CCR7*, *IL7R* and *LRRN3*) and early memory differentiation status, such as *TCF7* and *LEF1*. The dominant gene expression pattern of post-infusion CARPALL CAR-T cells was defined on a continuum of granzyme gene expression (Fig. 3c). Across post-infusion timepoints, CAR-T cells were skewed toward either higher *GZMH* and *GZMB* expression or higher *GZMK* expression. CAR-T populations that were defined by higher expression of *GZMK* additionally expressed genes related to effector (*LTB*), memory (*CD27* and *IL7R*) and activation (*CD28*) functions, whereas *GZMH*^+^*GZMB*^+^ cells expressed *FGFBP2* and *ZEB2*. Unlike the other patients in this study, with one exception (P09), most CAR-T cells at late timepoints expressed *GZMK*. In non-CAR-T cells, the *GZMH/B*-*GZMK* pattern of expression was also observed; however, CAR-T cells expressed *GZMK* to much higher levels (Extended Data Fig. 6a). The most recurrent and strongest markers of late CAR-T cells generated a persisting CAR-T signature that was delineated by the expression of bona fide immune-related genes, such as *TIGIT* and *GPR183*, as well as genes with unknown or emerging roles in immune biology (Fig. 3d and Extended Data Fig. 6b,c). The latter genes include *FXYD2*, *HMOX1, DENND2D* and *ISG20* (see Supplementary Table 5 for full gene signatures). The top marker of this population of cells was *FXYD2*, which encodes a modulator of the Na^+^/K^+^ ATPase channel. Of note, *FXYD2* was one of the transcripts expressed in functionally cryptic CD34^low^CD3^−^ CD4^+^CD8^−^ intrathymic T progenitors that have been described in the human thymus^10^. In aggregate, our data reveal that, within and across patients, thousands of CAR-T cells converge on a double-negative cellular phenotype that displays a common and distinct gene signature.

**Fig. 4:**
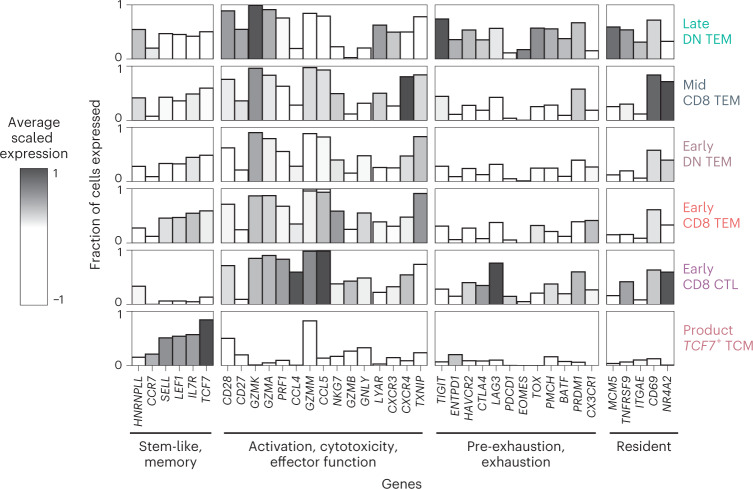
CAR-T cell immunophenotyping. Bar plots show custom gene modules that functionally characterize CAR-T cells. The height of the bar refers to the fraction of cells per cell type that express the gene. Higher bars indicate that more cells of that cell type are expressing that gene. The shading refers to the average scaled expression of those genes for that cell type. Darker shading indicates that the expression of that gene is above the average expression of that gene across all cell types. All CAR-T cells (product, early, mid and late) from all patients (*n* = 10) are analyzed. DN, double-negative; TCM, central memory; TEM, effector memory.

**Extended Data Fig. 7:**
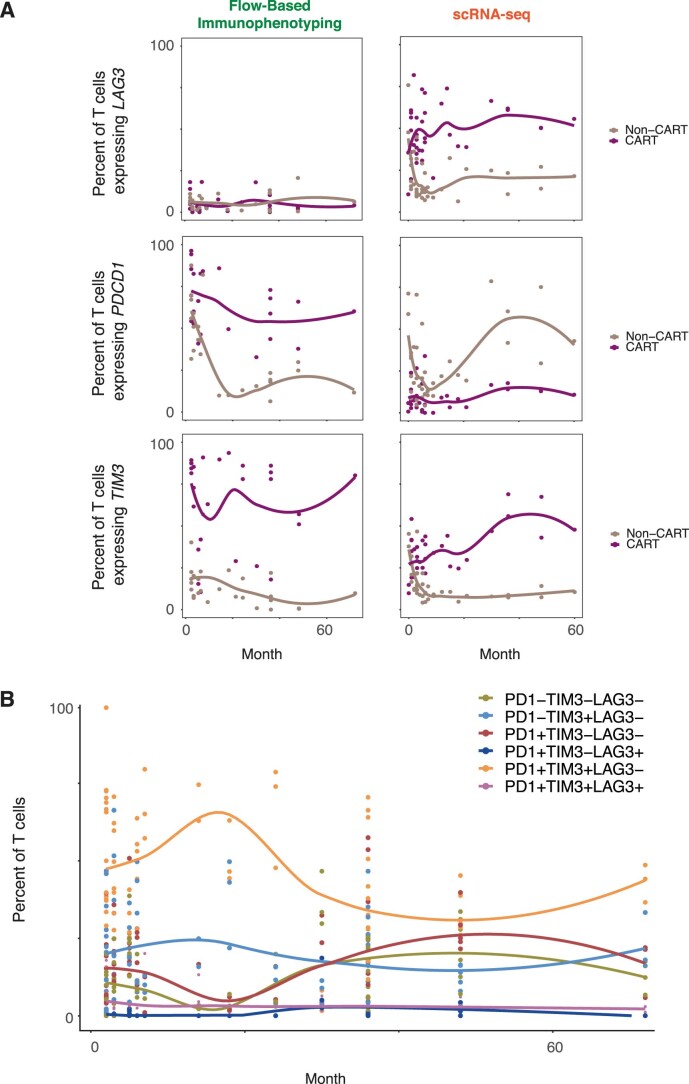
Exhaustion panel expression and flow-based immunophenotyping. **(a)** Trajectory scatter plots show the percent of T cells expression for *LAG3*, *PDCD1*, and *TIM3* across time for flow-based immunophenotyping (left) and single-cell RNA sequencing (scRNA-seq) data. Ribbons refer to the 95% confidence level interval for predictions using a ‘loess’ model to fit the line. **(b)** Trajectory scatter plots show the percent of T cells expression for the combination of *LAG3*, *PDCD1*, and *TIM3* across time for flow-based immunophenotyping. Ribbons refer to the 95% confidence level interval for predictions using a ‘loess’ model to fit the line.

In recent years, the classical dogma of a dichotomy between memory and exhausted T cells has been challenged with the description of functionally active memory cells that bear an imprint of prior exhaustion^11^. One of the most highly expressed genes in the persistence signature was the exhaustion marker *TIGIT*. We, therefore, assessed the co-expression of exhaustion markers in our CAR-T cells. We found that late CAR-T cells expressed canonical co-inhibitory receptors, such as *HAVCR2* and *LAG3*, but to a lesser extent *PDCD1* (Fig. 4). We, therefore, interrogated the gene and matched protein expression (flow cytometry) of these mediators related to exhausted and precursor exhausted T cells (Extended Data Fig. 7a). Precursor exhausted T cells have been isolated in human cancer, where their presence has been associated with response to immunotherapy. Like precursor exhausted T cells, late CAR-T cells bore expression of relevant effector genes, such as *GZMK* and *PRF1*, as well as transcription factors associated with T cell exhaustion, including *TOX*^12–14^*, NFATC1* (ref. ^15^)*, BATF*^16^ and *PRDM1* (ref. ^17^). Although exhibiting features of exhaustion, the late CAR-T cells did not appear terminally differentiated, as supported by low expression of *B3GAT1* (CD57) and by expressing no more than two exhaustion markers by flow cytometry (Extended Data Fig. 7b). Unlike CAR-T cells within the products that we evaluated, late CAR-T cells did not express high levels of *TCF7*, which orchestrates a state of memory stemness in precursor exhausted T cells in other contexts^18^. However, instead, there was robust expression of *JUN*, an AP-1-associated transcription factor that can mediate the reversal of T cell exhaustion and maintenance of cells with stem cell memory properties^19^. Thus, although long-persisting CAR-T cells did not exactly phenocopy precursor exhausted T cell populations described previously, this cell type would best describe their effector memory, exhaustion-imprinted status determined both transcriptionally and by flow cytometry.

### Polyclonal population structures of persisting CAR-T cells

**Fig. 5:**
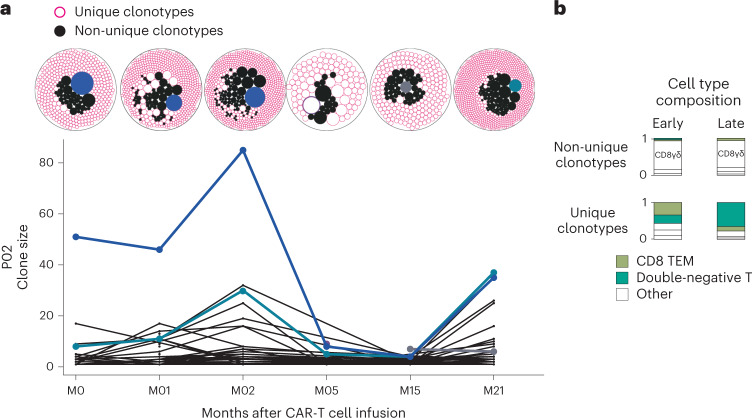
Population structures of CAR-T cells. **a**, Representative sample (P02) illustrating the changes in clonal architecture of CAR-T cells over time. Packed circle plots show the size of each clonotype. Filled-in black circles represent clonotypes that are not unique, as they are observed across timepoints. Conversely, pink donut circles represent clonotypes that are unique to that timepoint (and not observed across time). Blue/purple/gray colored circles represent the dominant clonotype at that timepoint that corresponds with the clonal trajectories below. **b**, Cell type composition stacked bar plots demonstrate the shift in cell type abundances between early and late timepoints and between unique and non-unique clonotypes. TEM, effector memory.

**Extended Data Fig. 8:**
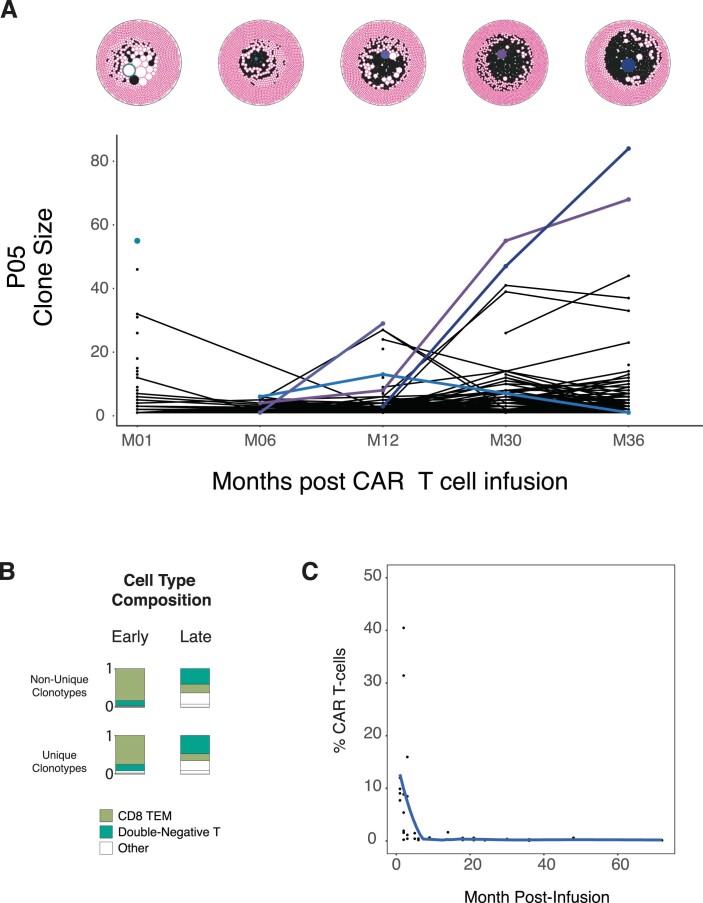
Clonal structure of CAR-T cells. **(a)** Clonal trajectory illustrates the changes in clonal architecture of CAR-T cells over time across a second representative patients with more than 3 timepoints sequenced. Packed circle plots show the size of each clonotype. Filled-in black circles represent clonotypes that are not unique, as they are observed across timepoints. Conversely, pink donut circles represent clonotypes that are unique to that timepoint (and not observed across time). Blue/purple/grey coloured circles represent the dominant clonotype at that timepoint that correspond with the clonal trajectories below. **(b)** Cell type composition stacked bar plots demonstrate the shift in cell type abundances between early timepoints and late timepoints between unique and non-unique clonotypes (right). (**c)** Trajectory scatter plots quantify data from all patient samples for CAR expression across time.

Within each patient, we had observed that, irrespective of T cell subset, thousands of cells converged on the same transcriptional state at late timepoints, raising the question of whether expansions of specific clones underpinned this functional convergence. We, therefore, interrogated TCR sequences of CAR-T cells and obtained readouts from 88 of 89 samples with concomitant gene expression data (Extended Data Fig. 1 and Supplementary Table 1). We found that the vast majority of cells across timepoints harbored unique clonotypes not observed at other timepoints. This indicates that the underlying gene pool remained sufficiently diverse to preclude the capture and tracking of individual clones (Fig. 5a and Extended Data Fig. 8a,b). An important consideration of this analysis is that the frequency of CAR-T cells diminishes over time, such that, by late timepoints, the frequency is as little as 0.05% of total CD3^+^ cells in circulating blood (Extended Data Fig. 8c). Nevertheless, if the population structure were monoclonal, we would capture the same clone on each blood draw. Of the few trackable clonotypes, the top 10 clonotypes at early timepoints remained among the relative majority at later timepoints but decreased in frequency over time. We observed an extensive variability in cell type composition among clonotypes, irrespective of whether they were unique or observed across timepoints. Clonotypes that were observed across timepoints were predominantly CD8^+^ T cells, whereas unique clonotypes tended to be double-negative T cells (Fig. 5b and Extended Data Fig. 8a,b). For two patients (P02 and P01), for whom we have infusion product TCR data, we were able to track 1.7% and 0.5% of clones across from infusion products to 2 years and 5 years, respectively. In aggregate, these clonal structures indicated that, at all timepoints, CAR-T cell populations were genetically diverse, consistent with insertion site analyses previously performed on CARPALL CAR-T cells^20^. In particular, there was no evidence of the dominance of one or more clones at late timepoints. Overall, these findings indicate that functional convergence of the persistence signature was not driven by clonal expansion.

### Evaluation of the persistence signature across T cells

**Fig. 6:**
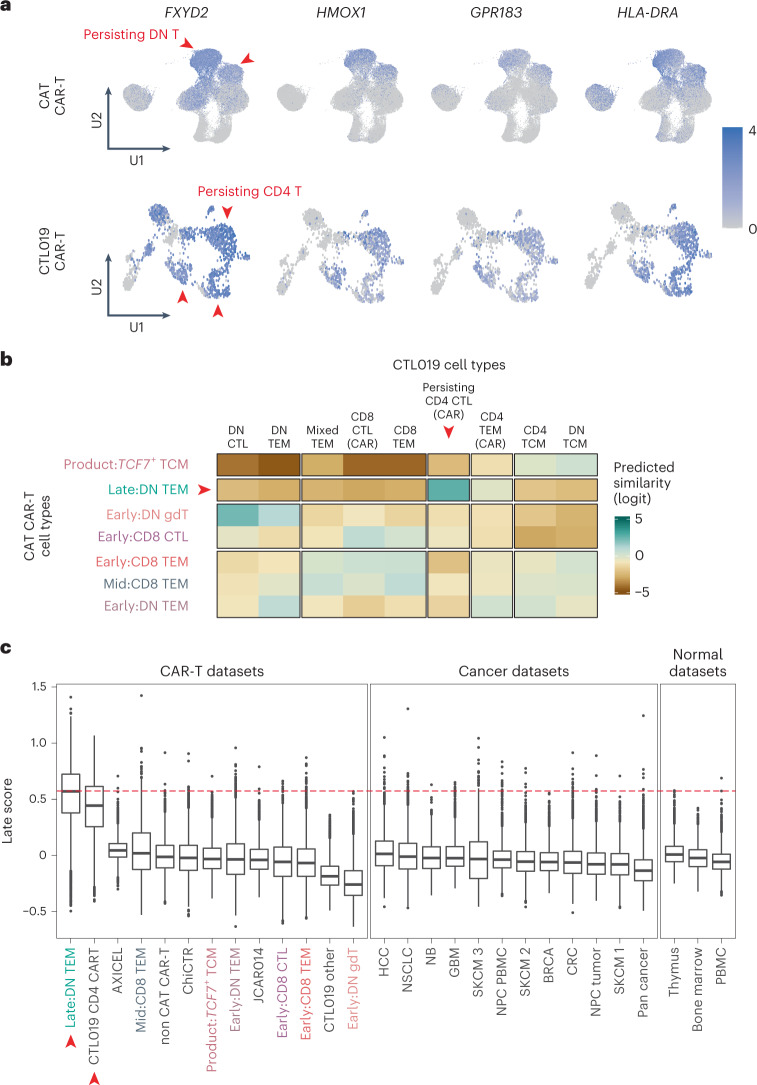
Evaluating the persisting transcriptional signature across T cells, including adult long-lived CAR-T cells. **a**, UMAP plots show expression of the strongest markers of the late-persisting CAR-T signature. Red arrows highlight persisting CAR-T cells between the CARPALL (CAT CAR-T) and CTL019 studies. **b**, Heat map demonstrates logistic regression cell-to-cell matching. CTL019-persisting CD4 CAR-T cells match strongly to late double-negative CAT CAR-T cells from the CARPALL study, as indicated by the red arrows. **c**, Box plots show the per-cell distribution of the late score as defined by the late-persisting CAR-T signature. Scores are shown for CAR-T, cancer and normal datasets. Publicly available datasets analyzed in this study are described in Supplementary Table 6. The red line represents the median of the late double-negative (DN TEM) cells from the CARPALL study. Colored cell types refer to CARPALL CAR-T cells. Red arrows indicate persisting CAT CAR-T cells from CARPALL and persisting CTL019 cells. Box plots show the first quartile (the lower end of the box) and the third quartile (the upper other end of the box) as well as the median values (center line) per dataset. The ‘whiskers’ extend from the ends of the box to a maximum and minimum of 1.5 times the interquartile range beyond the box. Outliers are shown as dots. AXICEL, axicabtagene ciloleucel CAR-T cells (infusion products); BRCA, breast cancer; ChiCTR, BCMA CAR-T (ChiCTR, 1800017404); CRC, colorectal cancer; DN, double-negative; GBM, glioblastoma; HCC, hepatocellular carcinoma; JCAR014, Fred Hutchinson Cancer Center CAR-T cells; NB, neuroblastoma; NPC, nasopharyngeal carcinoma; NSCLC, non-small lung cell carcinoma; SKCM, skin cutaneous melanoma (1 = Li; 2 = Yost; 3 = Jerby-Anon); TCM, central memory; TEM, effector memory. The numbers of cells and samples used in this figure are described in Supplementary Table 6.

As we had observed a transcriptional convergence of CAR-T cells across thousands of cells within and across patients, we speculated that the persistence signature may be pervasive across different CAR-T cell products. To date, one further single-cell transcriptomic study of persistent CAR-T cells has been reported—of two adult patients with CLL treated with anti-CD19 CAR-T cells (CTL019 cells) that have persisted for one decade thus far^7^. We interrogated CAR-T cell data from these two patients by assigning a persistence signature score to each cell (the AddModuleScore function in Seurat^21^). Remarkably, the module was expressed in CTL019-persisting CD4 CAR-T cells in almost its entirety (17/22 genes) (Fig. 6a). To compare our CARPALL CAR-T cell signal with CTL019 cells in an unbiased, quantitative manner, we used a method of cell-to-cell matching based on logistic regression^22^. We found that the strongest match of persisting CTL019 CD4^+^ CAR-T cells was to persisting double-negative T cells in the CARPALL data (Fig. 6b). It should be noted that persisting CTL019 cells were primarily derived from patient 1 (541/959, 56%), although, reassuringly, the persistence signature was also evident in a small number of cells from patient 2 (40/959, 4%). Overall, the similarity of persisting CARPALL and CTL019 CAR-T cells was not confined to gene sets but extended to the entire transcriptome.

**Extended Data Fig. 9:**
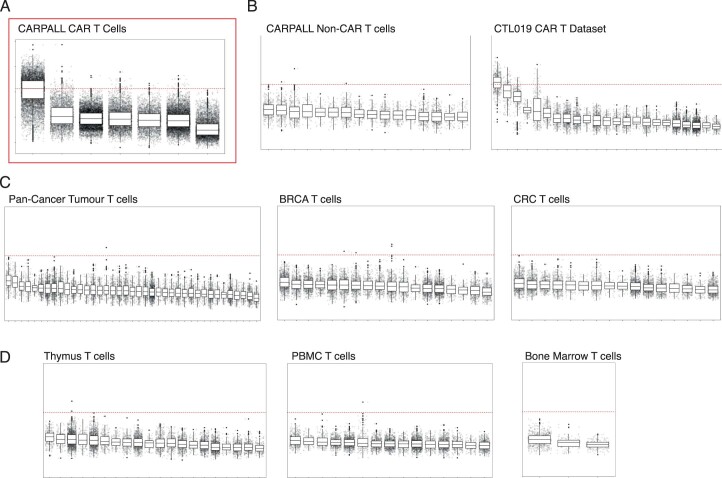
Evaluation of the persisting CAR-T cell signature across T-cells: expanded view. Boxplots show the per cell distribution of the late score as defined by the late, persisting CAR-T signature. Scores are shown for representative datasets encompassing (**a**) the CARPALL CAR-T cells, (**b**) CARPALL Non-CAR-T cells, CTL019 CAR-T cells, (**c**) Tumour-infiltrating T cells, and (**d**) T cells from normal datasets. The red dashed line represents the median of the late double-negative (DN TEM) cells from the CARPALL study. Across datasets, the x-axis ticks refer to Seurat-based clusters per dataset, with the exception of the CARPALL CAR-T cell dataset where the x-axis refers to annotated cell types. Box plots show the first quartile (the lower end of the box) and the third quartile (the upper other end of the box), as well as the median values (centre line) per dataset. The ‘whiskers’ extend from the ends of the box to a maximum and minimum of 1.5 times the interquartile range beyond the box. Outliers are shown as dots. BRCA = Breast Cancer, CRC=Colorectal Cancer, PBMC = Peripheral Blood Mononuclear Cells. Publicly available datasets analysed in this study and the number of cells and samples used in this figure are described in Supplementary Table 6.

We then questioned whether the persistence signature of CAR-T cells may have a physiological correlate. To this end, we scored T cells from a variety of healthy tissues, other CAR-T studies to date^5,6,23^ and cancer cell single-cell atlases, including normal peripheral blood^8^, human fetal bone marrow^24^, human fetal thymus^25^ and as many as 16 types of human cancers^26–34^, including tumors that are considered to be immunogenic and had long-term response to immune checkpoint inhibitors (for example, lung cancer and melanoma) (see Supplementary Table 6 for datasets analyzed). We were unable to detect T cells harboring the persistence signature at an appreciable frequency in any one tissue, barring occasional cells (Fig. 6c and Extended Data Fig. 9). The median frequency across tissues was −0.04 (−0.6 to 1.4). These observations indicate that the CAR-T cell persistence signature is rarely found in other biological contexts.

## Discussion

A lack of CAR-T cell persistence leading to CD19^+^ relapse is the main cause of therapy failure after licensed CAR-T cell therapy for ALL^35,36^ and contributes to relapse in other B cell malignancies, such as myeloma^37^. Therefore, a key question of CAR-T cell biology is why some cells persist whereas others perish. With this knowledge, we might better understand how to select patients, modify treatment phasing and optimize manufacturing protocols to support greater persistence and improve outcomes. To date, robust biomarkers of persistence have not been identified and can be validated only after directly demonstrating successful long-term persistence in patients. A key requirement of this is to systematically examine the biological status of long-lived CAR-T cells. Currently, there is a paucity of these datasets, as persisting CAR-T cells have been probed in only a very limited number of patients to date^7^. As such, we generated a single-cell RNA sequencing (scRNA-seq) dataset of cells from 10 patients with B-ALL treated with a CD19-targeting CAR-T cell product. Although our work represents, to our knowledge, the largest study of persisting single CAR-T cell transcriptomes, it still represents a modest cohort. Our key finding of a recurrent transcriptional state representing persistence is unlikely to be affected by the size of the cohort. Within each individual, every long-lived CAR-T cell represents a biological replicate of the signal. Accordingly, our finding has been reproduced multiple times within patients and has then been validated across individuals, including in the different clinical context of CLL. However, the size of our study precludes our ability to capture nuances of CAR-T cell transcription as well as any associations between CAR-T cell states and clinical subsets of patients, for which larger studies are required.

We found that late-persisting CAR-T cells mainly comprised a population that did not express CD8-α or CD4 co-receptors transcriptionally or via surface expression. In healthy individuals, double-negative cells typically comprise a minor population of all T cells, and we observed similar proportions in non-CAR T-cells from the same patient. In general, there was a steady reduction in CD8^+^ CAR-T cells over time, which matched a progressive increase in double-negative populations. This contrasts the long-lived CAR-T cells from adult CAR-treated patients with CLL, where double-negative CAR-T cell populations were noted at earlier timepoints and, on further investigation, were determined to be γδ T cells. Although γδ T cells were also observed in four patients in our cohort, they did not contribute to early CAR-T cell populations in the other patients. In our cohort, the predominance of double-negative CAR-T cells was particularly noted at later timepoints in all patients. We verified that, at the later timepoints, double-negative T cells were not contributed to by γδ CAR T cells or CAR NK cells. Late CAR-T cells in both cohorts showed evidence of an activated, proliferative and effector status with strong expression of *GZMA* and *GZMK*. Because the double-negative phenotype observed in late-persisting CAR-T cells is reminiscent of early thymocyte differentiation and the fact that we noted high expression levels of *GPR183*, an oxysterol receptor that provides survival and migratory signals to thymocytes and CD4^+^ T follicular helper cells^38^, we hypothesized a link between persisting CAR-T cells and thymic cell development. Ultimately, we found that the transcriptional status of the late, double-negative CAR-T cells did not map to any thymocyte subset in T cell development but, rather, to mature T cells.

Late-persisting CAR-T cells did not conform to quiescent early memory T cell populations but expressed genes associated with effector function and an activated state. These cells also maintained their proliferative capacity. In keeping with recent reports of precursor exhausted T cells bearing the hallmark of activation, late CAR-T cell populations expressed markers and transcription factors associated with exhaustion, including *TOX*^12–14^ and *BATF*^39^, among others. With reference to this highly activated status, one might speculate that these are circulating effector cells differentiated from rarer memory precursor populations after exposure to antigen. As these patients had no evidence of circulating B cells or existing CD19-expressing hematogones in the bone marrow, it is plausible that these cells were activated as the result of an emerging CD19-expressing hematogone population. However, the existence of minute central or stem cell memory CAR-T cells at this time-point may count against this hypothesis.

Late-persisting CAR-T cells, although activated and imprinted with markers of exhaustion, did not express *FOXO3* or *B3GAT1*, which are associated with terminal differentiation in the context of exhaustion. Instead, they expressed markers associated with memory-like characteristics, including *Jun*, *BCL2* and *IL7R*. Thus, they more closely matched precursor exhausted T cell populations as have been described in chronic viral infections^11,39,40^, cancer-infiltrating T cells^41,42^ and early post-infusion of CD19 CAR-T cells. Unlike previous reports of precursor exhausted T cell populations, however, they did not express high levels of *TCF7* (refs. ^39,40^) or *FOXO1* (refs. ^43,44^), confounding the suggested centrality of such transcription factors in driving long-lived CAR-T cell persistence and overcoming terminal exhaustion. In a previous report, c-Jun overexpression was sufficient to restore antigen responsiveness, memory function and long-term proliferative capacity in CAR-T cells exhausted due to tonic CAR signaling. The high expression of *JUN* in most late-persisting CAR-T cells in this study points to a plausible mechanism for long-lived persistence in these cells. Overall, our data instead support that, although previously exhausted CAR-T cells may indeed give rise to long-persisting populations, the predominance of transcription factors driving memory status is likely specific to the characteristics of the CAR, disease and model. Despite some similarities to precursor exhausted T cells, the persistence module was rarely expressed by T cells in a range of pathophysiological contexts. Within the signature, there were a number of genes with little-known roles in T cells, including *FXYD2*, *DENND2D* and *HMOX1*. Overall, further work is needed to elucidate their function in T cells and how they may contribute to persistence.

A key finding of this study was a transcriptional signature of persistence that was reproducible across thousands of cells in every patient with long-lived CAR-T cells and durable anti-B-ALL responses. This signature was not identified when interrogating non-CAR-T cells from the same patients, T cell populations in normal development, T cells from a range of cancer datasets or other CAR-T cell studies. The persistence signature and underlying cell state were detected in an independent dataset of long-lived CAR-T cells from adults with CLL who had received a different CD19 CAR-T cell product. Of note, persisting CAR-T cells from the independent dataset were cytotoxic CD4 T cells with oligoclonal population structures, in contrast to double-negative CAR-T cells that were polyclonal, as reported in this study. These differences could arise from the different techniques used for TCR analyses (integration site analysis versus single-cell TCR analysis), the number of patients evaluated or the fundamental differences in the CAR product. Although we noted these differences among CAR-T cells at different timepoints, the late CAR-T cell signature that we defined here was reproducible across both studies, indicating that it may represent a surrogate marker of longevity. Although this falls short of an easily measured biomarker of persistence with which to test CAR-T cell products, this understanding brings us a step closer to identifying such assays. That the transcriptional status noted was so pervasive in long-persisting CAR-T cells lends weight to the possibility that the signature may not only be a surrogate marker of longevity but, notably, may also provide a basis to investigate underlying cell-intrinsic or cell-extrinsic factors that drive CAR-T cell persistence. Given the data presented here, the longevity of CAR-T cells is likely not based on clonal selection and expansion. Rather, it is possible that the ongoing interplay with the environment shapes the resultant phenotype of long-lived CAR-T cells and supports functional diversity. With this knowledge, we will be primed in our ability to engineer this key characteristic into CAR-T cell therapies for hematological malignancies of the future.

## Methods

### Sample acquisition, ethics and patient consent

Data from this study were generated from patients enrolled in the CARPALL study (NCT02443831). CARPALL was a multi-center, non-randomized, open-label, phase 1, single-stage clinical study designed to evaluate the safety, efficacy and response of CD19 CAR-T cells in children and young adults (≤24 years of age) with high-risk relapsed CD19^+^ malignancies. Patient data were collected at Great Ormond Street Hospital (GOSH) and the University College London (UCL)-GOSH Institute of Child Health (ICH), and laboratory data were generated in the study central laboratories at GOSH, UCL-GOSH ICH as well as the Sanger Institute. Patient recruitment occurred from 2016 to 2019. Data collection, sequencing and analysis were from 2016 to 2023. The study protocol and outcomes are available here: http://www.ctc.ucl.ac.uk/TrialDetails.aspx?Trial=116&term=carpall. Key clinical factors for this cohort are described in Supplementary Table 7. All patients who took part in this study were diagnosed with B-ALL. Written informed consent was obtained from all patients or their parents/guardians before study entry. Patients did not receive compensation for participation in the study. Patient sex was reported by patients or parents and confirmed upon (external) examination. Study results do not apply to any one sex or gender. Sex or gender were not considered in the study design, as all children and young aduts with high-risk B-ALL, independent of sex/gender, were considered. The sex of patients was noted, and this is described in the table of patient characteristics (Supplementary Table 7). This trial was approved by the UK Medicines and Healthcare Products Regulatory Agency (clinical trial authorization no. 20363/0361/001). Ethical approval was obtained from the London–West London Gene Therapy Advisory Committee (GTAC) Research Ethics Committee (REC ref. no. 16/LO/0283). Note that the CARPALL study initially used monospecific low-affinity CD19 CAR-T cells for therapy of B-ALL; however, a study amendment allowing investigation of dual CD19 and CD22 CAR targeting is currently displayed on the ClinicalTrials.gov website. Historic versions of this trial before November 2020 can be viewed using the following link: https://clinicaltrials.gov/ct2/history/NCT02443831 (compare any version before November 2020). The analyses included here were not pre-specified in the clinical trial protocol.

### Flow cytometry

**Extended Data Fig. 10:**
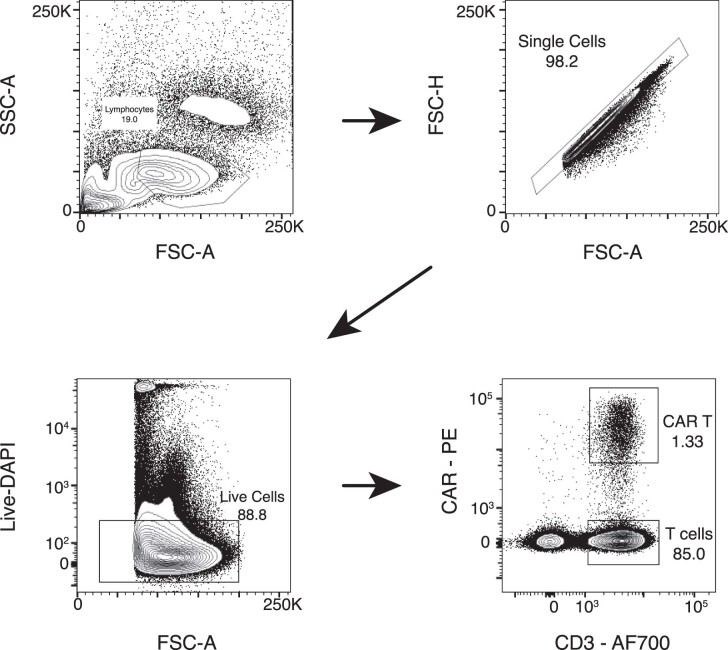
Flow cytometry gating strategy for CD3^+^ CAR^+^ T cells sorting. SSC = side scatter, FSC = forward scatter. Fluorochromes: FITC = Fluorescein isothiocyanate, PE = R-phycoerythrin, APC = Allophycocyanin.

CAR-T cells were isolated from either fresh peripheral blood or cryopreserved aliquots of the infusion product (IP), peripheral blood mononuclear cells (PBMCs) or bone marrow mononuclear cells (BMMCs). For fresh peripheral blood, PBMCs were isolated via density gradient centrifugation with Lymphopure (BioLegend). For cryopreserved samples, aliquots were rapidly thawed and washed in complete RPMI (10% FCS and 1% L-glutamine, Gibco). Flow cytometry was performed with a BD LSR II and cell sorting with a FACSAria III (BD Biosciences). Data analysis was performed using FlowJo version 10 (Tree Star) or FACS DIVA 8.0.1. Expression of CAR was detected by a CAR anti-idiotype antibody (bespoke product, Evitria, 1/200) and goat anti-rat IgG PE antibody (Poly4054, BioLegend, 1/400). The following reagents were used for phenotypic analysis of CAR-T cells: PD-1 BV421 (EH12.2H7, BioLegend, 1/20), CD45RA BV510 (HI100, BD Biosciences, 1/100), Lag3 BV605 (11C3C65, BioLegend, 1/20), TCRgd BV650 (B1, BD Biosciences, 1/20), CD127 BV711 (HIL-7R-M21, BD Biosciences, 1/20), CD4 BV784 (SK3, BioLegend, 1/100), CD25 VioBright FITC (4E3, Miltenyi Biotec, 1/100), Tim3 PECF594 (7D3, BD Biosciences, 1/20), CD8 PerCP-Cy5.5 (SK1, BioLegend, 1/40), CCR7 PE/Cy7 (G043H7, BioLegend, 1/40), CD95 APC (581, BioLegend, 1/10), CD3 AF700 (SK7, BioLegend, 1/40), CD27 APC/Cy7 (M-T271, BioLegend, 1/20), TIGIT BV605 (741182, BD Biosciences, 1/40), GPR183 PE/Dazzle594 (SA313E4, BioLegend, 1/40) and GZMK APC (GM26E7, BioLegend, 1/40). DAPI and Fixable Viability Dye eFluor 455UV (eBioscience) were used to discriminate viable cells. For intracellular markers, cells were fixed (Fixation Buffer, BioLegend) and permeabilized (Intracellular Staining Permeabilization Wash Buffer 10×, BioLegend) before staining. Human BD Fc Block (BD Biosciences) was used as a blocking reagent. Fluorescence minus one (FMO) controls were used to determine expression thresholds where required. The full list of antibodies can be found in Supplementary Table 8. The flow cytometry gating strategy for immunophenotyping can be found in Extended Data Fig. 10.

### CAR-T cell isolation and scRNA-seq using the 10x Chromium platform

Patient cells were harvested as described above for flow cytometry. Cryopreserved samples for 10x were rapidly thawed and washed with complete RPMI containing 50 U ml^−1^ of benzonase (Merck Life Science Limited). Cells were then stained with CAR anti-idiotype, followed by goat anti-rat IgG PE antibody and antibodies to CD3 APC (UCHT1, BioLegend, 1/20) and CD45 FITC (2D1, BioLegend, 1/20). DAPI was used to distinguish viable cells. CAR-T cells were isolated as CD45^+^CD3^+^CAR^+^ events in a live singlet leukocyte forward-scatter (FSC)/side-scatter (SSC) gate using a BD FACSAria III flow sorter. The flow cytometry gating strategy for CAR sorting can be found in Extended Data Fig. 10. CAR and non-CAR populations were sorted simultaneously and then immediately used downstream for the 10x workflow. Flow-sorted cells (CAR and non-CAR) were loaded according to the standard protocol of the Chromium Single Cell 5′ Kit (v2 chemistry). A TCR single-cell library was subsequently prepared from the same cells with the Chromium Single Cell V(D)J Enrichment Kit. The 5′ gene expression library and the TCR single-cell library were pooled with a molar ratio 10:1 for sequencing on Illumina NovaSeq S4 with 28 × 90 bp, aiming for an average of 300,000 reads per cell for the 5′ gene expression library and 30,000 reads per cell for the TCR single-cell library.

### Raw sequencing data processing, data filtering and normalization

The raw scRNA-seq data were demultiplexed and mapped to reference genome GRCh38, with the CAT-scFv sequence inserted, using Cell Ranger (10x Genomics, version 5.0.0). To filter lower-quality cells, we removed any cell with fewer than 300 genes, fewer than 1,000 unique molecular identifiers (UMIs) or where more than 10% of the read counts were derived from the mitochondrial genome. We excluded nuclear mitochondrial genes, heat shock proteins and ribosomal genes from our analysis.

Feature counts for each cell were divided by the total counts for that cell and multiplied by 10,000, followed by natural-log transformation using log1p. Counts data were then scaled such that each feature will be centered to have a mean of 0 and an s.d. of 1 for each gene. Principal component analysis was performed using the top 2,000 highly variable genes, and data were grouped into clusters using a community detection finding algorithm taking the first 75 principal components as inputs. Using these principal components, we calculated a UMAP for data visualization and calculated clusters using the *k*-nearest neighbors approach with resolution parameter set to 1. This was performed using the Seurat package in R (R version 4.0.3 and Seurat version 4.0.6).

### Cluster annotation and multi-modal reference mapping

CAR-T cells were defined as cells sorted for CD3 and the CAR by flow cytometry and belonging to clusters expressing the ‘CAT-scFv’ gene. CAR-T cells were clustered separately and labeled with their timepoint bins: product (M0), early (M1−M3), mid (M4−M6) and late (M7−M60). Clusters were subsequently annotated using lymphoid markers (that is, *CD8A*, *CD8B* and *CD4*) and established markers of T cell states curated from literature (Extended Data Fig. 3 and Supplementary Table 2). To supplement cell type annotation, the PBMC multi-modal reference was downloaded and processed using the instructions from the vignette. CAR-T cells were projected into the multi-modal reference using the FindTransferAnchors() and MapQuery() functions available in Seurat.

### Differential gene expression and immunophenotyping of CAR-T cells

CAR-T cells were clustered separately at a global (across patients) level and per patient. Seurat’s FindAllMarkers() function was used to identify differentially expressed genes from cells across patients (global clustering) and within a patient using previously annotated cell types and timepoint bins (product, early, mid or late) as the label (that is, late: CD8 TEM). These analyses were performed using the two-sided Wilcoxon rank-sum test with Bonferroni multiple testing correction. Only genes with an average log_2_ fold change above 0.5 were considered. For the per-patient analysis, markers were tallied and ordered from most to least recurrent across labels (timepoint bin: cell type). With the exception of the product, where only two samples were available, markers were considered recurrent if present in more than two patients. Gene signatures were derived from the intersection of the top 20 recurrent (across patients) marker genes and the global markers. For immunophenotyping analysis presented in Fig. 4, gene modules were curated from literature. The average scaled expression and percentage of cells expressing the gene were determined using the input derived from the data slot of the DotPlot() function in Seurat and replotted as shaded bar plots.

### TCR analysis

Chromium 10x V(D)J single-cell sequencing data were mapped and quantified using the software package cellranger vdj (version 5.0.0) using the GRCh38 reference (vdj_GRCh38_alts_ensembl-5.0.0). The consensus annotation files were generated per sample and used for downstream analyses. Clonotypes were defined per experimental sample based on unique TCR VJ sequences and complementarity-determining region (CDR3) motifs. Basic TCR statistics, such as the number of clones and the distribution of lengths and counts, were computed using Immunarch (version 0.7.0). For clonal tracking analyses, entries with a single or more than two alpha or beta chain(s) were considered one clone. Clonal population circles were created using the ggraph and igraph packages in R (version 2.0.5 and version 1.2.6, respectively). Unique clonotypes were defined as cells with shared TCR alpha and beta sequences that were not observed across timepoints but were uniquely observed at only one timepoint within the patient. Conversely, non-unique clonotypes are cells with shared TCR alpha and beta sequences that are present across at least two timepoints within a patient. The population circle plots were created by defining a ‘root’ and specifying the clonotype names and sizes as ‘branches’ on the same level of the tree.

### Cell-to-cell matching: logistic regression

To determine the probability that the transcriptome of each CARPALL CAT CAR-T cell was similar to CTL019 (tisagenlecleucel) CAR-T cells from two adult patients with CLL^7^, logistic regression was used in R, as previously described^22,26,45,46^. CTL019 raw counts data were processed as described above, using the same parameters as the CARPALL dataset. CTL019 cells were re-annotated using marker-based approaches, as described above. We trained logistic regression models with CTL019 cells using our cell type annotation.

### Gene module scoring

Published datasets from CAR-T cells, cancer and normal development were downloaded, and T cells were identified using *CD3D* and *CD3E* expression. T cell partitioned datasets were randomly downsampled to 10,000 cells, if exceeding this threshold. T cell clusters were processed and re-clustered, as described above. Module scores were calculated using the AddModuleScore() function available in Seurat using Seurat clusters as labels (Louvain algorithm). The average expression level of each cell type (or cluster) was calculated on a single-cell level and then subtracted by the aggregated expression of control feature sets. Gene modules were defined based on differential gene expression of CAT CAR-T cells.

### Reporting summary

Further information on research design is available in the Nature Portfolio Reporting Summary linked to this article.

## Online content

Any methods, additional references, Nature Portfolio reporting summaries, source data, extended data, supplementary information, acknowledgements, peer review information; details of author contributions and competing interests; and statements of data and code availability are available at 10.1038/s41591-023-02415-3.

## Supplementary information

Reporting Summary

Supplementary Tables 1–8.

Supplementary Data Source Data for supplementary figures.

## Data Availability

Raw sequencing data produced in this study have been deposited at the European Genome-phenome Archive (accession number EGAD00001010018). These data are available under restricted access. Sequencing data requests will be reviewed by the Independent Data Monitoring Committee and the Trial Management Group of the CARPALL study and will be subject to patient confidentiality. After approval, a data access agreement with University College London (UCL) will be required. All requests for raw materials will be reviewed by UCL Business (UCLB) to verify whether the request is subject to any intellectual property or confidentiality obligations. All requests will be processed within 8 weeks. Processed data have been uploaded to Zenodo^47^. Publicly available datasets analyzed in this study are described in Supplementary Table 6. The GRCh38 reference genome was downloaded from the 10x Genomics website: https://support.10xgenomics.com/single-cell-gene-expression/software/release-notes/build. Source data are provided with this paper.

## Source data

Source Data Fig. 2 Source data for timecourse flow-based immunophenotyping.

## Code availability

We have included the source code used to generate the figures and tables presented in this analysis. Code used in this study can be found here: https://github.com/natedandy/CAT_CART_paper_2023. Logistic regression was performed using code found here: https://github.com/constantAmateur/scKidneyTumors.

## References

[1] Maloney KW (2020). Outcome in children with standard-risk B-cell acute lymphoblastic leukemia: results of Children’s Oncology Group trial AALL0331. J. Clin. Oncol..

[2] Maude SL (2014). Chimeric antigen receptor T cells for sustained remissions in leukemia. N. Engl. J. Med..

[3] Ghorashian S (2019). Enhanced CAR T cell expansion and prolonged persistence in pediatric patients with ALL treated with a low-affinity CD19 CAR. Nat. Med..

[4] Xu, X. et al. Mechanisms of relapse after CD19 CAR T-cell therapy for acute lymphoblastic leukemia and its prevention and treatment strategies. *Front. Immunol.* **10**, 2664 (2019). 10.3389/fimmu.2019.02664 PMC6863137 31798590

[5] Sheih A (2020). Clonal kinetics and single-cell transcriptional profiling of CAR-T cells in patients undergoing CD19 CAR-T immunotherapy. Nat. Commun..

[6] Deng Q (2020). Characteristics of anti-CD19 CAR T cell infusion products associated with efficacy and toxicity in patients with large B cell lymphomas. Nat. Med..

[7] Melenhorst JJ (2022). Decade-long leukaemia remissions with persistence of CD4^+^ CAR T cells. Nature.

[8] Hao Y (2021). Integrated analysis of multimodal single-cell data. Cell.

[9] Pizzolato G (2019). Single-cell RNA sequencing unveils the shared and the distinct cytotoxic hallmarks of human TCRVδ1 and TCRVδ2 γδ T lymphocytes. Proc. Natl Acad. Sci. USA.

[10] Lee, M. S., Hanspers, K., Barker, C. S., Korn, A. P. & McCune, J. M. Gene expression profiles during human CD4^+^ T cell differentiation. *Int. Immunol.* **16**, 1109–1124 (2004). 10.1093/intimm/dxh112 15210650

[11] Galletti G (2020). Two subsets of stem-like CD8^+^ memory T cell progenitors with distinct fate commitments in humans. Nat. Immunol..

[12] Khan O (2019). TOX transcriptionally and epigenetically programs CD8^+^ T cell exhaustion. Nature.

[13] Scott AC (2019). TOX is a critical regulator of tumour-specific T cell differentiation. Nature.

[14] Alfei F (2019). TOX reinforces the phenotype and longevity of exhausted T cells in chronic viral infection. Nature.

[15] Martinez GJ (2015). The transcription factor NFAT promotes exhaustion of activated CD8^+^ T cells. Immunity.

[16] Quigley M (2010). Transcriptional analysis of HIV-specific CD8^+^ T cells shows that PD-1 inhibits T cell function by upregulating BATF. Nat. Med..

[17] Shin H (2009). A role for the transcriptional repressor Blimp-1 in CD8^+^ T cell exhaustion during chronic viral infection. Immunity.

[18] Chen Z (2019). TCF-1-centered transcriptional network drives an effector versus exhausted CD8 T cell-fate decision. Immunity.

[19] Lynn RC (2019). c-Jun overexpression in CAR T cells induces exhaustion resistance. Nature.

[20] Biasco L (2021). Clonal expansion of T memory stem cells determines early anti-leukemic responses and long-term CAR T cell persistence in patients. Nat. Cancer.

[21] Tirosh I (2016). Dissecting the multicellular ecosystem of metastatic melanoma by single-cell RNA-seq. Science.

[22] Young MD (2018). Single-cell transcriptomes from human kidneys reveal the cellular identity of renal tumors. Science.

[23] Li X (2021). Single-cell transcriptomic analysis reveals BCMA CAR-T cell dynamics in a patient with refractory primary plasma cell leukemia. Mol. Ther..

[24] Jardine L (2021). Blood and immune development in human fetal bone marrow and Down syndrome. Nature.

[25] Park JE (2020). A cell atlas of human thymic development defines T cell repertoire formation. Science.

[26] Kildisiute G (2021). Tumor to normal single-cell mRNA comparisons reveal a pan-neuroblastoma cancer cell. Sci. Adv..

[27] Li H (2019). Dysfunctional CD8 T cells form a proliferative, dynamically regulated compartment within human melanoma. Cell.

[28] Yost KE (2019). Clonal replacement of tumor-specific T cells following PD-1 blockade. Nat. Med..

[29] Jerby-Arnon L (2018). A cancer cell program promotes T cell exclusion and resistance to checkpoint blockade. Cell.

[30] Azizi E (2018). Single-cell map of diverse immune phenotypes in the breast tumor microenvironment. Cell.

[31] Guo X (2018). Global characterization of T cells in non-small-cell lung cancer by single-cell sequencing. Nat. Med..

[32] Lee HO (2020). Lineage-dependent gene expression programs influence the immune landscape of colorectal cancer. Nat. Genet..

[33] Liu Y (2021). Tumour heterogeneity and intercellular networks of nasopharyngeal carcinoma at single cell resolution. Nat. Commun..

[34] Zheng L (2021). Pan-cancer single-cell landscape of tumor-infiltrating T cells. Science.

[35] Dourthe ME (2021). Determinants of CD19-positive vs CD19-negative relapse after tisagenlecleucel for B-cell acute lymphoblastic leukemia. Leukemia.

[36] van Waart H (2015). Effect of low-intensity physical activity and moderate- to high-intensity physical exercise during adjuvant chemotherapy on physical fitness, fatigue, and chemotherapy completion rates: results of the PACES randomized clinical trial. J. Clin. Oncol..

[37] Munshi NC (2021). Idecabtagene vicleucel in relapsed and refractory multiple myeloma. N. Engl. J. Med..

[38] Li J, Lu E, Yi T, Cyster JG (2016). EBI2 augments Tfh cell fate by promoting interaction with IL-2-quenching dendritic cells. Nature.

[39] Utzschneider DT (2020). Early precursor T cells establish and propagate T cell exhaustion in chronic infection. Nat. Immunol..

[40] Utzschneider DT (2016). T cell factor 1-expressing memory-like CD8^+^ T cells sustain the immune response to chronic viral infections. Immunity.

[41] Siddiqui I (2019). Intratumoral Tcf1^+^PD-1^+^CD8^+^ T cells with stem-like properties promote tumor control in response to vaccination and checkpoint blockade immunotherapy. Immunity.

[42] Miller BC (2019). Subsets of exhausted CD8^+^ T cells differentially mediate tumor control and respond to checkpoint blockade. Nat. Immunol..

[43] Delpoux A, Lai CY, Hedrick SM, Doedens AL (2017). FOXO1 opposition of CD8^+^ T cell effector programming confers early memory properties and phenotypic diversity. Proc. Natl Acad. Sci. USA.

[44] Michelini RH, Doedens AL, Goldrath AW, Hedrick SM (2013). Differentiation of CD8 memory T cells depends on Foxo1. J. Exp. Med..

[45] Khabirova E (2022). Single-cell transcriptomics reveals a distinct developmental state of *KMT2A*-rearranged infant B-cell acute lymphoblastic leukemia. Nat. Med..

[46] Custers L (2021). Somatic mutations and single-cell transcriptomes reveal the root of malignant rhabdoid tumours. Nat. Commun..

[47] Anderson, N. D. Transcriptional signatures associated with persisting CD19 CAR T-cells in children with leukaemia. 10.5281/ZENODO.7937878 (2023). 10.1038/s41591-023-02415-3 PMC10353931 37407840

