# Supplementary material for: Transcriptional signatures associated with persisting CD19 CAR-T cells in children with leukemia
Source: Nat Med. 2023 Jul 6;29(7):1700–9. doi: 10.1038/s41591-023-02415-3 (PMC10353931; doi:10.1038/s41591-023-02415-3)
Supplement: Supplementary file 1 — Reporting Summary [file 41591_2023_2415_MOESM1_ESM.pdf]

nature portfolio

Corresponding author(s): Sam Behjati and Sara Ghorashian

Last updated by author(s): May 16, 2023

Reporting Summary

Nature Portfolio wishes to improve the reproducibility of the work that we publish. This form provides structure for consistency and transparency in reporting. For further information on Nature Portfolio policies, see our Editorial Policies and the Editorial Policy Checklist.

Statistics

For all statistical analyses, confirm that the following items are present in the figure legend, table legend, main text, or Methods section.

n/a	Confirmed	
☐	☒	The exact sample size (*n*) for each experimental group/condition, given as a discrete number and unit of measurement
☐	☒	A statement on whether measurements were taken from distinct samples or whether the same sample was measured repeatedly
☐	☒	The statistical test(s) used AND whether they are one- or two-sided<br>*Only common tests should be described solely by name; describe more complex techniques in the Methods section.*
☐	☒	A description of all covariates tested
☐	☒	A description of any assumptions or corrections, such as tests of normality and adjustment for multiple comparisons
☐	☒	A full description of the statistical parameters including central tendency (e.g. means) or other basic estimates (e.g. regression coefficient) AND variation (e.g. standard deviation) or associated estimates of uncertainty (e.g. confidence intervals)
☒	☐	For null hypothesis testing, the test statistic (e.g. F, t, r) with confidence intervals, effect sizes, degrees of freedom and P value noted<br>*Give P values as exact values whenever suitable.*
☒	☐	For Bayesian analysis, information on the choice of priors and Markov chain Monte Carlo settings
☒	☐	For hierarchical and complex designs, identification of the appropriate level for tests and full reporting of outcomes
☒	☐	Estimates of effect sizes (e.g. Cohen's *d*, Pearson's *r*), indicating how they were calculated

Our web collection on statistics for biologists contains articles on many of the points above.

Software and code

Policy information about availability of computer code

Data collection	No software was used for data collection.
Data analysis	Flow data analysis was performed using FlowJo v10 (Tree Star, Inc., Ashland OR), or FACs DIVA 8.0.1. Single-cell analyses was performed using the Seurat package in R (R version 4.0.3, Seurat version 4.0.6). The raw scRNA-seq data were demultiplexed and mapped to reference genome GRCh38, with the CAT-scFv sequence inserted, using Cell Ranger (10x Genomics, version 5.0.0). Chromium 10× V(D)J single-cell sequencing data were mapped and quantified using the software package cellranger vdj (v5.0.0) using the GRCh38 reference (vdj_GRCh38_alts_ensembl-5.0.0). Basic TCR statistics, such as the number of clones and the distribution of lengths and counts, were computed using Immunarch (version 0.7.0). Clonal population circles were created using the ggraph and igraph packages in R (version 2.0.5 and version 1.2.6, respectively). Logistic regression was performed using custom code found here: similarity.R in https://github.com/constantAmateur/scKidneyTumors.

For manuscripts utilizing custom algorithms or software that are central to the research but not yet described in published literature, software must be made available to editors and reviewers. We strongly encourage code deposition in a community repository (e.g. GitHub). See the Nature Portfolio guidelines for submitting code & software for further information.

Data

Policy information about availability of data

All manuscripts must include a data availability statement. This statement should provide the following information, where applicable:

- Accession codes, unique identifiers, or web links for publicly available datasets
- A description of any restrictions on data availability
- For clinical datasets or third party data, please ensure that the statement adheres to our policy

Raw sequencing data produced in this study has been deposited at the European Genome-phenome Archive (accession number EGAD00001010018). This data is available under restricted access. Sequencing data requests will be reviewed by the Independent Data Monitoring Committee and Trial Management Group of the CARPALL study and will be subject to patient confidentiality. After approval, a data-access agreement with UCL will be required. All requests for raw materials will be reviewed by UCL Business (UCLB) to verify whether the request is subject to any intellectual property or confidentiality obligations. All requests will be processed within 8 weeks. Processed data has been uploaded to Zenodo. Publicly available datasets analysed in this study are described in Supplementary Table 6. The GRCh38 reference genome was downloaded from the 10X Genomics website: https://support.10xgenomics.com/single-cell-gene-expression/software/release-notes/build.

Human research participants

Policy information about studies involving human research participants and Sex and Gender in Research.

Reporting on sex and gender	Study results do not apply to any one sex or gender. Sex or gender were not considered in the study design, as all high risk B-ALL children and young adults, independent of sex/gender, were considered. The sex of patients was noted and these are described in the table of patient characteristics (Supplementary Table 7).
Population characteristics	Children and young adults (age 24 years or younger) with high risk/relapsed CD19+ haematological malignancy.
Recruitment	Children meeting the inclusion criteria were recruited at Great Ormond Street Hospital, University College Hospital and Manchester Royal Children's Hospital as part of a previously reported clinical trial. Written, informed consent was obtained from all patients or their parents/guardians prior to study entry. The present secondary investigation did not actively recruit any new patients.
Ethics oversight	The present study is an investigation using samples collected from an existing clinical trial. Ethical approval was obtained from the London West London & Gene Therapy Advisory Committee (GTAC) Research Ethics Committee (REC ref. no. 16/LO/0283).

Note that full information on the approval of the study protocol must also be provided in the manuscript.

Field-specific reporting

Please select the one below that is the best fit for your research. If you are not sure, read the appropriate sections before making your selection.

☒ Life sciences ☐ Behavioural & social sciences ☐ Ecological, evolutionary & environmental sciences

For a reference copy of the document with all sections, see nature.com/documents/nr-reporting-summary-flat.pdf

Life sciences study design

All studies must disclose on these points even when the disclosure is negative.

Sample size	Data from this study were generated from patients enrolled in the CARPALL study (NCT02443831). We performed detailed phenotyping by flow cytometry in 11 of 15 patients, and for ten children, sufficient CAR T-cells were obtained from serial time points for further interrogation by single-cell mRNA and TCR sequencing. Sample size calculation was not performed as samples were derived from whichever patients were enrolled in the study.
Data exclusions	No data were excluded
Replication	Single-cell RNA sequencing findings in this study were orthogonally validated using flow-based immunophenotyping and by comparing results to similar publicly available datasets. Experiments were not replicated and performed independently (each sample was sequenced once).
Randomization	Randomization is not relevant in this study, as we characterized long-lived CAR T-cells from children with leukemia. The study design does not include allocation of samples into experimental groups. All samples with sufficient material were sequenced and analyzed.
Blinding	Blinding was not relevant in this study as patients were not allocated to groups during data collection or analysis. The purpose of this study was to characterize long-lived CAR T-cells from children with leukemia.

Reporting for specific materials, systems and methods

We require information from authors about some types of materials, experimental systems and methods used in many studies. Here, indicate whether each material, system or method listed is relevant to your study. If you are not sure if a list item applies to your research, read the appropriate section before selecting a response.

Materials & experimental systems

n/a	Involved in the study
☐	☒ Antibodies
☒	☐ Eukaryotic cell lines
☒	☐ Palaeontology and archaeology
☒	☐ Animals and other organisms
☐	☒ Clinical data
☒	☐ Dual use research of concern

Methods

n/a	Involved in the study
☒	☐ ChIP-seq
☐	☒ Flow cytometry
☒	☐ MRI-based neuroimaging

Antibodies

Antibodies used

The following antibodies were used for identification and phenotypic analysis of CAR T cells in accordance with manufacturer's instructions or as indicated: CAR anti-idiotype antibody (bespoke product, Evitria, 1/200), PD-1 BV421 (EH12.2H7, Biolegend #329920, 1/20), CD45RA BV510 (HI100, BD #563031, 1/100), Lag3 BV605 (11C3C65, Biolegend #369324, 1/20), TCRgd BV650 (B1, BD #564156, 1/20), CD127 BV711 (HIL-7R-M21, BD #563165, 1/20), CD4 BV784 (SK3, Biolegend #344642, 1/100), CD25 VioBright FITC (4E3, Miltenyi #130-113-282, 1/100), Goat anti-Rat IgG PE (Poly4054, Biolegend #405406, 1/400), Tim3 PECF594 (7D3, BD #565560, 1/20), CD8 PerCP-Cy5.5 (SK1, Biolegend #344710, 1/40), CCR7 PE/Cy7 (G043H7, Biolegend #353226, 1/40), CD95 APC (581, Biolegend #305612, 1/10), CD3 AF700 (SK7, Biolegend #344822, 1/40), and CD27 APC/Cy7 (M-T271, Biolegend #356424, 1/20); TIGIT BV605 (741182, BD #747841, 1/40), GPR183 PE/Dazzle594 (SA313E4, Biolegend #368917, 1/40) and GZMK APC (GM26E7, Biolegend #370510, 1/40); CD45 FITC (2D1, Biolegend #368508, 1/20) and CD3 APC (UCHT1, Biolegend #300439, 1/20).

Validation

Primary antibodies were validated and titrated with appropriate antigen positive and negative controls in order to determine the optimal stain concentration for each test. CAR T cell detection with the CAR anti-idiotype antibody were validated against healthy donor controls and pure populations of CAR T cells in accordance with standard operating protocols in UK NEQAS and ISO accredited laboratories. The following list is an overview of the validations performed for each antibody:

Target ; Fluorochrome ; Clone ; Validation
PD-1 ; BV421 ; EH12.2H7 ; Resting and anti-CD3/CD28 activated PBMC measured at 24-, 48- and 72-hours post-activation
CD45RA ; BV510 ; HI100 ; Healthy donor PBMC T-cell population phenotyping
Lag3 ; BV605 ; 11C3C65 ; Resting and anti-CD3/CD28 activated PBMC measured at 24-, 48- and 72-hours post-activation
TCRgd ; BV650 ; B1 ; Healthy donor PBMC T-cell population phenotyping
CD127 ; BV711 ; HIL-7R-M21 ; Healthy donor PBMC T-cell population phenotyping
CD4 ; BV784 ; SK3 ; Healthy donor PBMC T-cell population phenotyping
CD25 ; VioBright FITC ; 4E3 ; Healthy donor PBMC T-cell population phenotyping
CAR anti-idiotype ; N/A ; Bespoke ; In vitro non-transduced and transduced CAR T cells with transgene distal 2A mCherry
Goat anti-rat IgG ; PE ; Poly4054 ; In vitro non-transduced and transduced CAR T cells with transgene distal 2A mCherry
Tim3 ; PECF594 ; 7D3 ; Resting and anti-CD3/CD28 activated PBMC measured at 24-, 48- and 72-hours post-activation
CD8 ; PerCP-Cy5.5 ; SK1 ; Healthy donor PBMC T-cell population phenotyping
CCR7 ; PE/Cy7 ; G043H7 ; Healthy donor PBMC T-cell population phenotyping
CD95 ; APC ; 581 ; Healthy donor PBMC T-cell population phenotyping
CD3 ; AF700 ; SK7 ; Healthy donor PBMC T-cell population phenotyping
CD27 ; APC/Cy7 ; M-T271 ; Healthy donor PBMC T-cell population phenotyping
TIGIT ; BV605 ; 741182 ; in vitro non-transduced and CAR T cells repetitively stimulated with CD3/CD28 dynabead and Nalm6 target cells
GPR183 ; PE/Dazzle594 ; SA313E4 ; Healthy donor PBMC T-cell population phenotyping and in vitro CAR T cells
GZMK ; APC ; GM26E7 ; Healthy donor PBMC T-cell population phenotyping and in vitro CAR T cells
CD45 ; FITC ; 2D1 ; Healthy donor PBMC T-cell population phenotyping
CD3 ; APC ; UCHT1 ; Healthy donor PBMC T-cell population phenotyping

Clinical data

Policy information about clinical studies
All manuscripts should comply with the ICMJE guidelines for publication of clinical research and a completed CONSORT checklist must be included with all submissions.

Clinical trial registration

NCT02443831

Study protocol

http://www.ctc.ucl.ac.uk/TrialDetails.aspx?Trial=116&term=carpall

Data collection

The present study is not a clinical study but uses samples from a previous clinical trial. Patient data were collected at Great Ormond Street Hospital (GOSH) and UCL-GOSH Institute of Child Health (ICH), whereas laboratory data were generated in the study central

	laboratories at GOSH, UCL-GOSH ICH, as well as the Sanger Institute. Patient recruitment occurred from 2016-2019. Data collection, sequencing, and analysis was from 2016-2023.
Outcomes	The present study is not a clinical trial, but uses samples from a previous clinical trial. Primary and secondary outcomes from the initial trial can be found in the above protocol. There were no primary or secondary outcomes reported in this manuscript.

Flow Cytometry

Plots

Confirm that:

☒ The axis labels state the marker and fluorochrome used (e.g. CD4-FITC).

☒ The axis scales are clearly visible. Include numbers along axes only for bottom left plot of group (a 'group' is an analysis of identical markers).

☒ All plots are contour plots with outliers or pseudocolor plots.

☒ A numerical value for number of cells or percentage (with statistics) is provided.

Methodology

Sample preparation	For flow cytometry-based immunophenotyping, patient cells were acquired from either fresh peripheral blood or cryopreserved aliquots of the infusion product (IP), peripheral blood mononuclear cells (PBMC) or bone-marrow mononuclear cells (BMMC). For fresh peripheral blood, PBMC were isolated via density gradient centrifugation with Lymphopure (Biolegend). For cryopreserved samples, aliquots were rapidly thawed and washed in complete RPMI (10% FCS and 1% L-glutamine, Gibco). Up to 10x10^6 cells were stained in 100ul of Cell Staining Buffer (Biolegend). For intracellular markers, cells were fixed (Fixation Buffer, Biolegend) and permeabilised (Intracellular Staining Permeabilization Wash Buffer 10X, Biolegend) prior to staining. Human BD Fc Block (BD) was used as a blocking reagent to reduce non-specific interactions.<br><br>For fluorescence-activated cell sorting, patient cells were acquired from either fresh peripheral blood or cryopreserved aliquots of the IP, PBMC or BMMC. Fresh peripheral blood was processed as described and cryopreserved samples were rapidly thawed and washed with complete RPMI containing 50U/mL of Benzonase Nuclease (Merck Life Science Limited). Up to 10x10^6 cells were stained in 100uL of Cell Staining Buffer (Biolegend). Human BD Fc Block (BD) was used as a blocking reagent to reduce non-specific interactions.
Instrument	Flow cytometry was performed with a BD LSR II (4-laser configuration; 355nm, 405nm, 488nm and 633nm) and cell sorting with a FACS Aria III (4-laser configuration; 405nm, 488nm, 561nm and 647nm) (BD Biosciences)
Software	Data analysis was performed using FlowJo v10 (Tree Star, Inc., Ashland OR), or FACs DIVA 8.0.1.
Cell population abundance	Cell population abundances are shown in the flow cytometry plots and tables of raw data.
Gating strategy	For immunophenotyping, CAR-T cells were isolated as viable CD3+/CAR+ events in a singlet leukocyte forward-scatter (FSC)/ side-scatter (SSC) gate<br><br>For cell sorting, CAR-T cells were isolated as viable CD45+/CD3+/CAR+ events in a singlet leukocyte forward-scatter (FSC)/ side-scatter (SSC) gate.

☒ Tick this box to confirm that a figure exemplifying the gating strategy is provided in the Supplementary Information.
